# Virulence determinants and toxin profile of methicillin resistant *Staphylococcus aureus* from commercial cheese in Bangladesh: A public health risk

**DOI:** 10.1371/journal.pone.0350222

**Published:** 2026-06-11

**Authors:** Muhammad Tafazzal Hossain, Fahmida Jahan Fahim, Sohel Rana, Nadia Sultana, Md. Sodor Uddin, Md Nazim Uddin, Monira Noor, Amina Khatun, Kazi Mohammad Ali Zinnah, Anzuman Ara, Ferdaus Mohd Altaf Hossain

**Affiliations:** 1 Department of Dairy Science, Faculty of Veterinary, Animal and Biomedical Sciences, Sylhet Agricultural University, Sylhet, Bangladesh; 2 Department of Microbial Biotechnology, Faculty of Biotechnology and Genetic Engineering, Sylhet Agricultural University, Sylhet, Bangladesh; 3 Department of Livestock Production and Management, Faculty of Veterinary, Animal and Biomedical Sciences, Sylhet Agricultural University, Sylhet, Bangladesh; 4 Department of Pathology, Faculty of Veterinary, Animal and Biomedical Sciences, Sylhet Agricultural University, Sylhet, Bangladesh; 5 Department Pathology, Faculty of Animal Science and Veterinary Medicine, Sher-e-Bangla Agricultural University, Sylhet, Bangladesh; 6 Department of Animal and Fish Biotechnology, Faculty of Biotechnology and Genetic Engineering, Sylhet Agricultural University, Sylhet, Bangladesh; Mekdela Amba University, ETHIOPIA

## Abstract

Cheese, a widely consumed dairy product, can be contaminated with *Staphylococcus aureus (S. aureus)*, a pathogen capable of producing toxins harmful to humans. Of particular concern is Methicillin-Resistant *S. aureus* (MRSA), which harbors antimicrobial resistance genes and secretes super-antigenic toxins. Present investigation aimed at determining the occurrence and characterizing MRSA in commercial cheese, to evaluate its potential public health risks. 120 cheese samples representing twelve commercial brands were collected in ten different batches over a six-month period in Sylhet, Bangladesh. The quality of the cheeses was evaluated and compared against the standards of Bangladesh Food Safety Authority (BFSA), European Union (EU), and Food Safety and Standards Authority of India (FSSAI). The occurrence of MRSA and its virulence factors were determined using standard microbiological and molecular techniques. Results revealed that 65% samples were positive for *S. aureus*, with staphylococcal load surpassing safety thresholds according to the above-mentioned standards. MRSA was detected in 30% of the samples, exhibiting resistance to multiple antibiotics. All the isolates showed resistance against penicillin, tetracycline, doxycycline, trimethoprim-sulfamethoxazole and azithromycin, whereas ceftaroline, norfloxacin, and levofloxacin exhibited intermediate level of sensitivity. Enterotoxin genes *SEa* and *SEc* were prevalent in 16.67% and 8.97% of isolates, respectively, while *TSST-1* gene was identified in 25.64% among the exfoliative toxin genes. Notably, 85.90% and 80.77% of the isolates exhibited biofilm formation based on the Congo Red and microtiter plate techniques, respectively, with significant percentages of biofilm associated regulatory genes *icaA* (73.08%), *icaD* (53.85%), *clfA* (78.21%)*, clfB* (61.54%)*,* and *fnbA* (69.23%). The substantial prevalence of *bla*_*CTX-M-2a*_ (88.46%) and *bla*_*TEM*_ (25.64%) highlights the significant public health risks associated with MRSA contamination in cheese. The high occurrence of *S. aureus* and the detection of multiple toxin-encoding genes further emphasize the need for strengthened monitoring and stricter control measures across the production and distribution chain to reduce contamination and ensure product safety.

## 1. Introduction

The growing consumption of dairy products, particularly cheese, emphasizes the critical intersection of food safety, public health, and One Health approach. This nexus is exemplified by the threat posed by *S. aureus*, a notorious pathogen known for its resistance to conventional antibiotics and its capacity to produce potent toxins, including super-antigenic properties [1,2]. *S. aureus* has been identified as a principal agent in food-related disease outbreaks globally [3]. The ability of *S. aureus* to contaminate and multiply in various food products underlines the critical risk it poses to food safety [4]. Staphylococcal food poisoning, a direct consequence of consuming contaminated food, is often associated with the manual handling of food under unsanitary conditions [3,5].

Cheese, particularly homemade varieties made primarily from raw milk and frequently produced under unsanitary conditions, has been identified as a potent source of food-borne pathogens [6,7]. Given its potential to cause food-borne illness, maintaining the microbiological safety of the product is vital for consumer health [8]. However, it has been observed that Bangladesh’s total sanitary maintenance of the production, processing, packaging, and distribution of milk do not satisfy the required level [9]. As a result, infections caused by *S. aureus*, particularly methicillin-resistant *S. aureus* (MRSA), are becoming a greater public health issue across the country [10,11].

A variety of extracellular toxins and virulence factors produced by *Staphylococcus aureus* have a role in the development of illness [12]. The most important virulence factor of *S. aureus* is resistance to antibiotics and its biofilm-forming capacity [13]. MRSA infections have proven to be one of the biggest issues with antibiotic treatment [14]. Antimicrobial resistance has emerged as a consequence of the widespread use of antimicrobial agents in veterinary and human medicine, coupled with other agricultural practices and animal husbandry [15].

Enterotoxins are important due to their thermal stability and resistance to inactivation by gastrointestinal proteases such as pepsin [16]. Staphylococcal enterotoxins (SEs) can act as super-antigens that cause unregulated activation of the immune response [17]. Thus, its presence in raw milk and dairy products raises serious concerns for health and the quality of traditional cheese production due to the presence of *SEa, SEb, SEc, SEd, SEe* toxins*,* and toxic shock syndrome toxin (*TSST-1*) [18].

Despite the growing consumption of dairy products in Bangladesh, including cheese, limited research has examined their microbiological safety, particularly with regard to *S. aureus* and its methicillin-resistant strains (MRSA). Within this context, there is a striking paucity of studies addressing the prevalence, antimicrobial resistance profiles, and virulence determinants of MRSA in commercial cheeses. While MRSA is globally recognized as a significant foodborne pathogen due to its ability to carry antimicrobial resistance genes and produce super-antigenic toxins [19,20], its presence and potential risks in Bangladesh’s cheese supply chain remain largely uncharacterized.

To address this knowledge gap, the present study focused on the detection and molecular characterization of MRSA in commercial cheeses in Bangladesh. Specifically, we sought to identify strains harboring genes encoding super-antigenic toxins, assess their antimicrobial resistance patterns, and evaluate their potential public health implications. By doing so, this research not only highlights the risks posed to consumers but also contributes to the broader discourse on antimicrobial resistance (AMR) within the food sector. The findings are expected to inform interventions that align with the One Health framework, integrating human, animal, and environmental health to strengthen food safety strategies in Bangladesh.

## 2. Materials and methods

### 2.1. Sample collection and processing

Over a six-month sampling period, 120 cheese samples from twelve commercially available brands were collected in ten batches from retail outlets in Sylhet, Bangladesh. Except for one brand produced from raw milk, all cheeses were manufactured from pasteurized milk. Samples were collected aseptically and transported under chilled conditions (4°C) to the laboratory for microbiological analysis. Strict precautions were maintained throughout sampling, transport, and handling to prevent self- and cross-contamination and ensure the accuracy of results.

### 2.2. Microbiological quality of cheeses

The quality of the cheese samples was evaluated and compared against the standards of Bangladesh Food Safety Authority (BFSA), European Union (EU), and Food Safety and Standards Authority of India (FSSAI). The microbiological evaluation comprises the total load of *Staphylococci* in each sample. For this, samples were homogenized in phosphate buffer saline (PBS) in 1:9 ratio and followed the 10-fold serial dilution method [21]. Aliquots of 100 µL from each dilution were spread onto Mannitol Salt Agar (MSA) (Oxoid, Thermo Fisher Scientific, UK) plates and incubated at 37 °C for 18–24 hours. Petri plates which had only characteristic yellow colonies were selected and calculated as CFU/gm of cheese [22].

### 2.3. Detection of *Staphylococcus aureus* and MRSA

For detection of *S. aureus* and MRSA, samples were streaked onto MSA plates, specific for *Staphylococcus* genus*,* and incubated for 18–24 hours at 37°C. From each plate, the colonies that best exhibited the properties were chosen for all downstream works. Gram staining and biochemical tests such as catalase and coagulase tests were carried out was for morphological confirmation. DNA was extracted from pure culture using total genomic DNA extraction kit (Thermo Fisher Scientific, UK), and used as a template for molecular detection of *S. aureus* and MRSA using specific primer sets [23,24]. PCR products were separated on 2% of agarose gel using 100 bp DNA ladder (Thermo Fisher Scientific, UK), and DNA amplicons were visualized with ethidium bromide. All the primer sets, product sizes, cycling conditions and corresponding references were given in the table (S1 Table).

### 2.4. Antibiotic sensitivity testing

The antibiotic sensitivity test was performed using the Kirby-Bauer disk diffusion method following Clinical and Laboratory Standards Institute (CLSI) guidelines 2025. Briefly, the test was performed on Mueller-Hinton agar (MHA) (Oxoid, Thermo Fisher Scientific, UK) with an inoculum equivalent to 0.5 McFarland corresponding to a cell concentration of approximately 10^8^ CFU/ml (OD_600nm_ = 0.1). Bacterial inoculum (100 μL) was spread onto the agar plate, antibiotic discs were placed and kept into an incubator for 18–24 h at 37 °C. The zone diameter was measured using a slide caliper in millimeter (mm) and categorized as sensitive, intermediate, and resistant according by CLSI 2025 (S2 Table).

### 2.5. Phenotypic and genotypic characterization of biofilm formation

The biofilm-forming ability of *S. aureus* isolates was assessed phenotypically using the Congo Red Agar (CRA) and tissue culture plate (TCP) methods, following our previously described protocol [25]. For the CRA assay, the medium was prepared by supplementing 1 L of blood agar with 0.8 g Congo red (Oxoid, Thermo Fisher Scientific, UK). Fresh *S. aureus* cultures were streaked onto the plates and incubated at 37 °C for 24–48 h. The appearance of black colonies indicated slime (biofilm) production, whereas red colonies denoted non-producers.

For the TCP assay, overnight cultures were grown in trypticase soy broth (TSB) (Oxoid, Thermo Fisher Scientific, UK) at 37 °C for 24 h and adjusted to approximately 10⁸ CFU/mL (0.5 McFarland standard). The cultures were then serially diluted 10-fold in fresh TSB, and 200 µL of each diluted suspension was added to triplicate wells of 96-well flat-bottomed microtiter plates. Wells containing only TSB served as negative controls. After incubation at 37 °C for 24 h, wells were washed 3–5 times with sterile phosphate-buffered saline (PBS) to remove planktonic cells, fixed with 95% ethanol for 5 min, and stained with 1% (v/v) crystal violet. Excess stain was removed by rinsing with sterile distilled water, and plates were air-dried. The optical density (OD) was measured at 570 nm using a spectrophotometer. Isolates were classified as strong (OD_570 nm_ ≥ 1), moderate (0.1 ≤ OD_570 nm_ < 1), or non-biofilm producers (OD_570 nm_< 0.1). Further, the isolates were subjected to PCR amplification to detect biofilm-associated regulatory genes (*icaA, icaB, icaC, icaD, clfA, ClfB, and fnbA*) using specific primer sets (S1 Table).

### 2.6. Detection of virulence and antibiotic resistant genes

All the isolates were screened for staphylococcal enterotoxins (*SEa, SEb, SEc, SEd, SEe*), exfoliative toxins (*eta, etb, tsst*), and antibiotic resistant genes (*CTX-M-2a, CTX-M-1, CTX-M, OXA-1, TEM, CMY, SHV, NDM-1*) using multiplex PCR. PCR products were separated and DNA was visualized following the procedures described above. The primer sequences, expected amplicon sizes, cycling conditions and corresponding references are presented in S3 Table.

### 2.7. Statistical analysis and data visualization

All data obtained in this study were compiled in Microsoft Excel 365 and analyzed using GraphPad Prism (version 9.3.1; San Diego, CA, USA) and R-Studio (version 4.3.1; Boston, MA, USA). Graphical representations were generated using GraphPad Prism, while the heatmap of phenotypic antibiotic resistance profile was constructed with the “pheatmap” package in R. Data are expressed as mean ± SEM, and differences were considered statistically significant at P < 0.05 using t-test.

## 3. Results

### 3.1. Microbial quality of cheese

**Fig 1:**
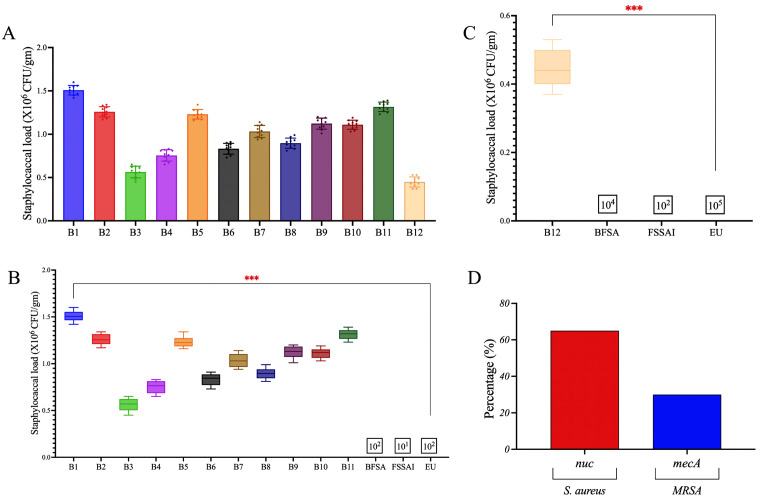
Microbial load and occurrence of *Staphylococcus aureus* and MRSA in commercial cheese samples.

The total Staphylococcal count in the analyzed cheese samples was considerably higher than the permissible limits established by the Bangladesh Food Safety Authority (BFSA), the Food Safety and Standards Authority of India (FSSAI), and the European Union (EU). The average bacterial load was 1.01 × 10⁶ CFU/g, ranging from 0.37 × 10⁶ to 1.6 × 10⁶ CFU/g. In cheeses produced from pasteurized milk, Staphylococcal loads exceeded the BFSA and EU limits by approximately 10⁴-fold and the FSSAI standard by 10⁵-fold. Conversely, in the cheese made from raw milk, microbial counts were 10-, 100-, and 10⁴-fold higher than the permissible levels set by the EU, BFSA, and FSSAI, respectively. These findings indicate poor microbiological quality across all brands and suggest potential lapses in hygienic practices during production and handling (Fig 1A-C).

### 3.2. Occurrence of *S. aureus* and MRSA

All cheese samples (120) were subjected to cultural, biochemical, and molecular analyses to determine the presence of *S. aureus* and methicillin-resistant *S. aureus* (MRSA). Based on typical colony morphology on selective media, subsequent biochemical characterization and PCR targeting the *nuc* gene confirmed *S. aureus* in 65% (78/120) of the samples (S1 Fig). Among these, 30% (36/120) harbored the *mecA* gene, indicating the presence of MRSA strains (S2 Fig). These findings highlight a substantial contamination rate of *S. aureus* in commercial cheeses and the circulation of methicillin-resistant strains within the dairy supply chain (Fig 1D, S4 Table).

### 3.3. Antibiotic sensitivity

**Fig 2:**
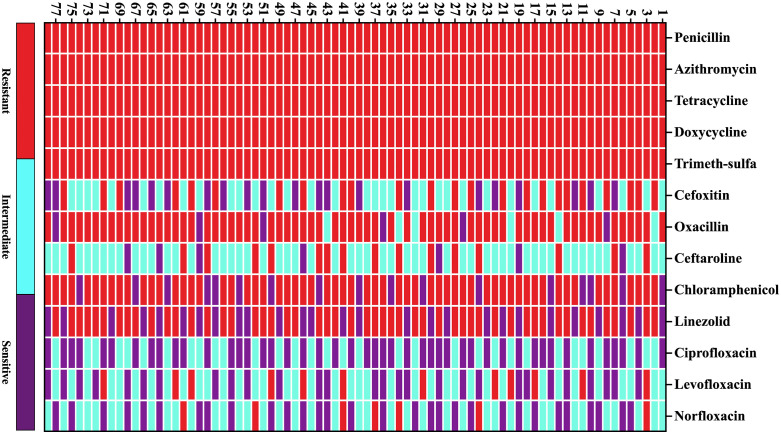
Heatmap representation of antimicrobial susceptibility patterns of *Staphylococcus aureus* isolates.

**Fig 3:**
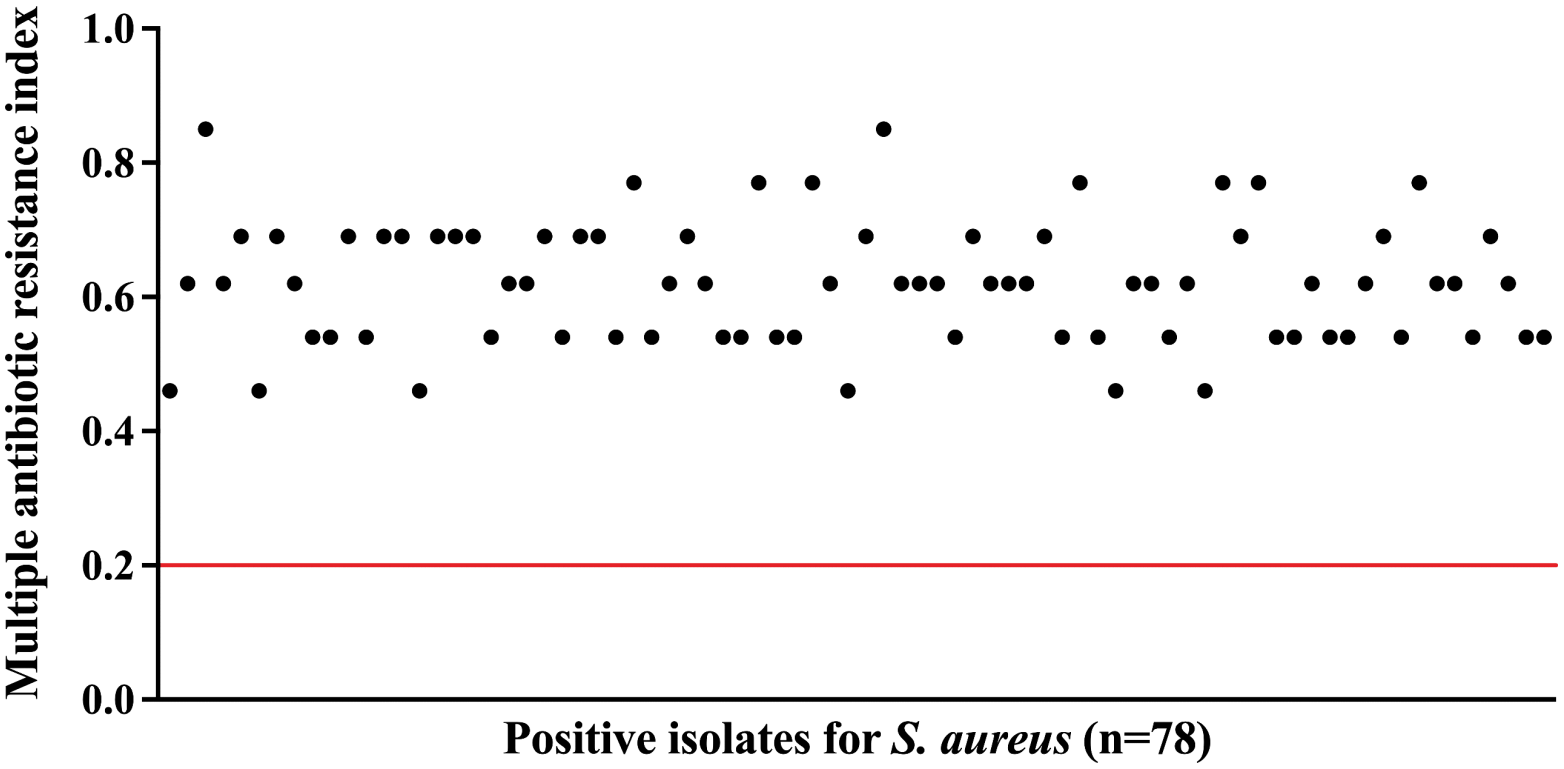
Multiple antibiotic resistance (MAR) index of *Staphylococcus aureus* isolates.

Antimicrobial susceptibility testing was performed against thirteen antibiotics following CLSI (2025) guidelines. All *S. aureus* isolates exhibited complete (100%) resistance to penicillin, tetracycline, doxycycline, trimethoprim-sulfamethoxazole and azithromycin (Fig 2). In contrast, intermediate level of resistance was observed for ceftaroline, levofloxacin, and norfloxacin. Notably, 58.33% of the isolates remained sensitive to ciprofloxacin, suggesting it as one of the few antibiotics retaining efficacy against the tested strains. The majority of isolates showed resistance to widely used antibiotics as tetracycline, doxycycline, and penicillin. All isolates exhibited MAR index values exceeding 0.2, indicating substantial antimicrobial pressure and potential environmental contamination (Fig 3).

### 3.4. Biofilm production

**Fig 4:**
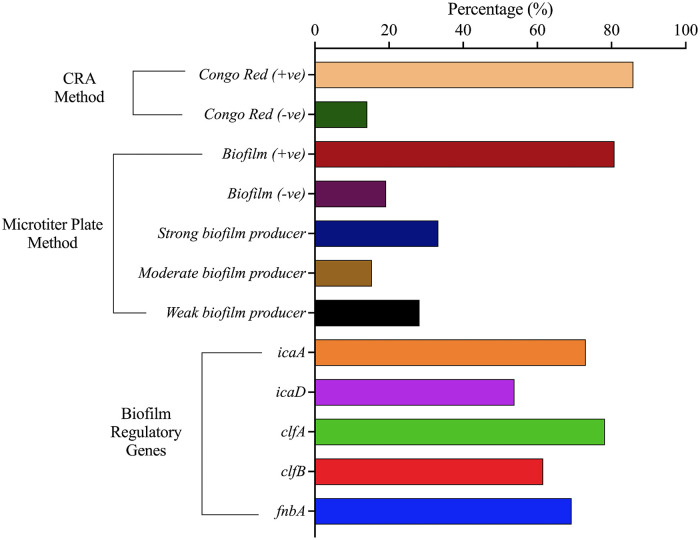
Phenotypic and genotypic characterization of biofilm formation in *Staphylococcus aureus* isolates.

Based on the CRA method, 85.90% isolates produced black colonies of dry consistency, indications of slime producing strains. According to microtiter plate test, 80.77% isolates exhibited biofilm forming ability, whereas the percentages of strong, intermediate and weak biofilm producers were 33.33%, 15.38%, and 28.21%, respectively. Among the biofilm regulatory genes, *icaA, icaD, clfA, clfB,* and *fnbA* were the most prevalent. PCR results revealed that the presence of *icaA, icaD, clfA, clfB,* and *fnbA* were 73.08%, 53.85%, 78.21%, 61.54%, and 69.23%, respectively (Figs 4, S3, S5 Table). However, *icaB* and *icaC* were not found in our experiments.

### 3.5. Virulence and antibiotic-resistant genes

**Fig 5:**
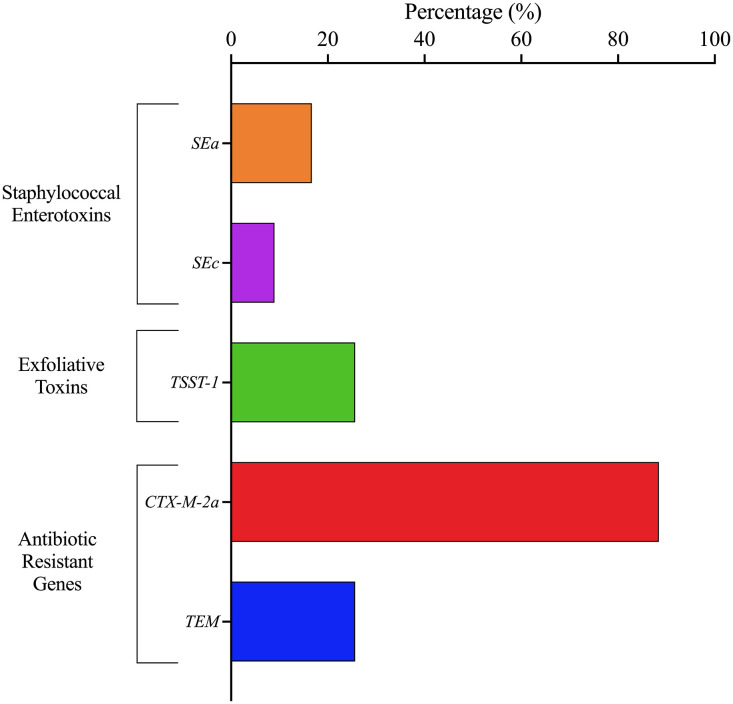
Distribution of virulence and antibiotic-resistance genes among *Staphylococcus aureus* isolates.

All isolates were screened for virulence genes associated with toxin production and for antibiotic resistant determinants. Results demonstrated that among the staphylococcal enterotoxin genes, *SEa* and *SEc* were detected in 16.67% and 8.97% of isolates. Regarding exfoliative toxins, *TSST-1* was identified in 25.64% of isolates, while *etA and etB* were not detected. Screening of antibiotic resistant genes (ARGs) revealed the presence of *bla*_*CTX-M-2a*_ and *bla*_*TEM*_ in 88.46% and 25.64% of isolates, respectively; other ARGs were tested absent (Figs 5, S4–S7, S4 Table).

## 4. Discussions

The increasing incidence of foodborne diseases globally, attributed significantly to pathogens like *S. aureus*, necessitates a renewed focus on microbial hazards in food items, especially those consumed widely like cheese. *S. aureus*, capable of thriving across a wide environmental spectrum, presents a formidable challenge to food safety due to its resilience and the virulence of certain strains that produce enterotoxins and other super-antigens. These toxins, recognized for their thermal stability and ability to resist gastrointestinal degradation, can cause severe gastrointestinal and systemic symptoms, posing heightened risks, particularly to vulnerable populations.

In the present study, commercial cheese samples from Sylhet exhibited *S. aureus* contamination levels well above regulatory microbial safety thresholds set by national and international authorities. The average bacterial load (~ 1.01 × 10⁶ CFU/g) significantly exceeds common safety standards, underscoring a serious public health concern. The heavy contamination observed in our samples aligns with global evidence indicating frequent contamination of dairy products with *S. aureus*. A recent meta-analysis reported that approximately 42.8% of cheese samples worldwide were positive for *S. aureus* [26]. In similar studies, soft and artisanal cheeses have shown contamination levels ranging from 5.5 × 10¹ to 7.6 × 10⁶ CFU/g, with mean counts around 4.0 × 10⁵ CFU/g (27). For example, in one study on soft cheeses, 68.9% of samples were contaminated, with some exceeding 10⁵ CFU/g, a level considered hazardous due to the risk of enterotoxin production [27]. These findings are consistent with our results and reinforce the notion that dairy products, particularly soft cheeses, provide favorable environments for *S. aureus* survival and growth.

The prevalence of *S. aureus* detected in our study (65%) is considerably higher than global pooled estimates, where approximately 40–45% of cheese samples are reported to contain the organism [26]. MRSA was present in 30% of all samples, a proportion notably higher than the generally low MRSA detection rates (<5%) reported in dairy products globally [28]. Comparative regional studies show variable results depending on sample type and setting. Local Bangladeshi surveys of raw milk and market milk products have documented the presence of *S. aureus* in milk and dairy items, but few have reported cheese-specific prevalence at the scale observed here. In a study of local markets, Nusrat et al., 2015 found *S. aureus* in 8 (20%) out of 40 cheese samples, whereas Haque et al., 2018 confirmed *S. aureus* presence in cheese, with 57 (79.17%) isolates identified across 72 dairy product samples [10,29]. Studies from neighboring South Asian settings and Pakistan have also reported substantial *S. aureus* contamination in certain cheese and milk samples, indicating that soft cheeses and informal dairy chains are commonly at risk [30]. The higher prevalence in our study may reflect product type (soft cheese readily supports staphylococcal growth), post-pasteurization contamination, inadequate hygienic practices during handling and retail, or the use of contaminated raw materials or starter cultures. These factors have been implicated in previous studies as key drivers of *S. aureus* contamination in dairy chains [31].

Our isolates exhibited 100% resistance to five antibiotics- penicillin, tetracycline, doxycycline, trimethoprim-sulfamethoxazole and azithromycin, while showing intermediate resistance to ceftaroline, levofloxacin and norfloxacin, and a moderate sensitivity to ciprofloxacin (≈58.33%). These resistance patterns align with several regional reports demonstrating widespread resistance of *S. aureus* from dairy sources to commonly used antibiotics. A recent study from Bangladesh on mastitis-associated *S. aureus* isolates reported high resistance to tetracycline (~74.5%), oxacillin (55.9%) and trimethoprim-sulfamethoxazole (30.0%) [32]. Another nationwide investigation observed penicillin and amoxicillin resistance as almost universal among dairy-derived *S. aureus*, with frequent resistance to tetracycline and azithromycin as well [33]. The consistency of high tetracycline and β-lactam resistance suggests that these antibiotics remain poor choices for treating dairy-associated staphylococcal contamination in this region.

Conversely, moderate sensitivity to fluoroquinolones particularly ciprofloxacin in our isolates resonates with reports from both Bangladesh and neighboring regions. In a northern Bangladesh dairy-herd study, approximately 70% of *S. aureus* isolates were susceptible to ciprofloxacin, and cefoxitin susceptibility was also observed in a majority of isolates [33]. Similarly, dairy-associated *S. aureus* from parts of South Asia and the Indian subcontinent frequently display lower resistance rates against fluoroquinolones and chloramphenicol compared to older drug classes [34]. The breadth of resistance seen in our cheese-derived isolates especially the complete resistance to multiple first-line antibiotics underscores serious public-health implications. Considering that similar multidrug-resistant phenotypes are found in clinical and animal-origin *S. aureus* isolates in Bangladesh, the possibility of transmission through the food supply is concerning.

The majority of isolates in this study exhibited strong biofilm phenotypes, with 85.90% positive on Congo Red Agar and 80.77% showing biofilm formation by microtiter assay; strong, intermediate and weak producers accounted for 33.33%, 15.38% and 28.21%, respectively. These phenotypic rates are higher than several regional reports but fall within ranges reported globally, where food-related *S. aureus* biofilm positivity by microtiter assays often ranges from ~40–80% [35]. Genotypically, the high prevalence of adhesion and biofilm-associated genes in our isolates *icaA* (73.08%), *icaD* (53.85%), *clfA* (78.21%), *clfB* (61.54%) and *fnbA* (69.23%) concurs with studies that link these loci to strong biofilm phenotypes in food and clinical isolates [36]. Compared with limited Bangladeshi data, where *icaA* prevalence has been reported at lower frequencies (e.g., ~ 42–50% in some food/milk surveys), our findings suggest an elevated burden of biofilm-capable strains in the sampled cheeses [37]. The absence of *icaB* and *icaC* in our panel highlights genetic heterogeneity among dairy *S. aureus* isolates and ind*ica*tes that multiple, sometimes *ica*-independent, mechanisms (surface adhesins such as *Clf* and *FnB* proteins) likely contribute to biofilm formation in these strains. This widespread biofilm capacity has practical implications: biofilm-forming *S. aureus* are more persistent on food-contact surfaces and more tolerant to sanitizers and antimicrobials, thereby increasing the risk of contamination and persistence within the cheese production chain [38].

In this study, a subset of *S. aureus* isolates carried classic enterotoxin genes (*SEa* 16.67%, *SEc* 8.97%) and toxic-shock associated locus (*TSST-1* 25.64%), while *etA* and *etB* were absent. The detection of these virulence genes indicates that a proportion of cheese borne isolates possess the genetic capacity to produce toxins associated with staphylococcal food poisoning and severe toxin-mediated disease. However, it is important to emphasize that PCR detection of toxin genes indicates potential rather than proven toxin expression; phenotypic assays (e.g., enterotoxin ELISA) would be required to confirm active toxin production.

Compared with previous work, the frequency and distribution of enterotoxin genes vary by geography and sample type. Global and regional surveys report *SEa* as one of the most commonly detected enterotoxin genes in food and dairy strains, but relative frequencies differ (for example, *SEa* prevalence has been reported at ~20–30% in some surveys, while other studies have found *SEc* or non-classical enterotoxin genes to predominate depending on the source) [39]. In Bangladesh, molecular surveys have likewise documented heterogeneous enterotoxin profiles among *S. aureus* isolates, with some studies reporting *SEc* as frequent and multiple-gene carriage common; our findings of detectable *SEa* and *SEc* therefore align with prior regional observations but suggest somewhat lower carriage rates than some published series [40]. The relatively high prevalence of *TSST-1* (25.64%) in our isolates is noteworthy. While *TSST-1* is not universally common among food isolates, several studies have reported appreciable *TSST-1* frequencies in both clinical and food-associated *S. aureus,* underscoring the potential for toxin-mediated systemic effects if such strains contaminate foods consumed by vulnerable individuals [41]. On the antibiotic-resistance side, the near-ubiquity of *bla*_*CTX-M-2a*_ (88.46%) and the detection of *bla*_*TEM*_ in ~25.64% of isolates are striking and of major public-health concern. *bla*_*TEM*_ encodes β-lactamases that commonly confer resistance to penicillin and related agents, consistent with phenotypic β-lactam resistance reported in many regional studies of dairy and clinical *S. aureus* isolates. The high *bla*_*TEM*_ carriage we observed concords with prior reports from Bangladesh and neighboring settings that implicate widespread β-lactamase-mediated resistance in food-linked staphylococci [42]. The presence of *bla*_*CTX-M-2a*_, a gene classically associated with extended-spectrum β-lactamases (ESBLs) in Enterobacteriaceae within *S. aureus* isolates is less commonly reported but has been documented in a few studies, raising the possibility of interspecies horizontal gene transfer mediated by mobile genetic elements. Such acquisitions would broaden the β-lactam resistance repertoire of staphylococci and complicate therapeutic options and decontamination efforts. Regardless of the precise origin, the co-occurrence of virulence determinants and β-lactamase genes in food-borne *S. aureus* underscores the food chain’s role as a reservoir and conduit for clinically relevant resistance determinants [43].

Overall, our findings reveal a high burden of *S. aureus* contamination in commercial cheeses, characterized by multidrug resistance, strong biofilm-forming ability, and the presence of key virulence and toxin-encoding genes. The co-occurrence of these traits highlights a significant public-health risk and underscores the potential for transmission of pathogenic and antimicrobial-resistant strains through the dairy supply chain. This study was limited by its sample size and geographic scope, as cheese samples were collected only from Sylhet and may not represent national contamination patterns. Additionally, toxin gene detection was based solely on PCR without phenotypic confirmation of enterotoxin expression, and whole-genome sequencing was not performed, restricting deeper insights into strain relatedness, mobile genetic elements, and AMR transmission dynamics. Strengthened hygiene practices, routine microbiological screening of dairy products, and stricter regulation of antimicrobial use in food-animal production are urgently needed. Enhanced surveillance integrating phenotypic and genotypic monitoring within a One Health framework should be prioritized to reduce contamination risks and limit the spread of resistant *S. aureus* strains.

## 5. Conclusion

This study demonstrates that commercial cheeses available in Sylhet are heavily contaminated with *S. aureus*, including methicillin-resistant strains, at levels far exceeding national and international safety standards. The high prevalence of multidrug resistance, strong biofilm-forming ability, and the presence of key virulence and toxin-associated genes highlight the significant public health risks associated with these products. The detection of *bla*_*TEM*_ and *bla*_*CTX-M-2a*_ further underscores the potential for dissemination of clinically relevant resistance determinants through the food chain. Collectively, these findings indicate substantial gaps in hygiene, post-processing handling, and antimicrobial oversight within the dairy sector. Strengthening sanitary practices, enforcing routine microbiological surveillance, and integrating One Health–based interventions are essential steps to reduce contamination, limit the spread of resistant *S. aureus*, and ensure safer dairy products for consumers in Bangladesh.

## Supporting information

S1 Table Primer sets for detecting S. aureus, MRSA and biofilm regulatory genes. (DOCX)

S2 Table Antibiotics used in sensitivity test against S. aureus isolates. (DOCX)

S3 Table Primer sets for detecting virulence and antimicrobial resistance genes. (DOCX)

S4 Table Distribution of S. aureus, MRSA, virulence and antibiotic resistant genes. (DOCX)

S5 Table Phenotypic and genotypic characterization of biofilm production. (DOCX)

S1 Fig Gel image of amplified *nuc* gene of *S. aureus.* (TIF)

S2 Fig Gel image of amplified *mecA* gene. (TIF)

S3 Fig Gel image of amplified *icaA* and *icaD* genes. (TIF)

S4 Fig Gel image of amplified exfoliative and enterotoxin genes. (TIF)

S5 Fig Gel image of amplified exfoliative and enterotoxin genes. (TIF)

S6 Fig Gel image of amplified exfoliative and enterotoxin genes. (TIF)

S7 Fig Gel image of amplified antimicrobial resistance genes of *S. aureus.* (ZIP)
