# Supplementary material for: Virulence determinants and toxin profile of methicillin resistant Staphylococcus aureus from commercial cheese in Bangladesh: A public health risk
Source: PLoS One. 2026 Jun 11;21(6):e0350222. doi: 10.1371/journal.pone.0350222 (PMC13257977; doi:10.1371/journal.pone.0350222)
Supplement: S1 Table — (DOCX) [file pone.0350222.s001.docx]

## Table S1: Primer sets for detecting *S. aureus*, MRSA and biofilm regulatory genes

|  | **Target genes** | **Nucleotide Sequences (5’-3’)** | **Amplicon size (bp)** | **References** |
| --- | --- | --- | --- | --- |
| *Staphylococcus aureus* | *nuc* | F- GACTCGGTTTAGTTCACAGA  R- CACACGCTGACGCTGACCA | 280 | (Oliveira et al., 2016) |
| MRSA | *mecA* | F-CCTAATGTCATTCGCTCTGC  R-CGGGTATAGCTACTGTCACC | 286 | (Adhikari et al., 2017) |
| Biofilm-producing genes | *icaA* | F-AAAATCGATGGTAAAGGTTGGC  R- AGTTCTGCAGTACCGGATTTGC | 188 | (Anzabi & Shayegh, 2021) |
|  | *icaB* | F-AGAATCGTGAAGTATAGAAAATT  R-TCTAATCTTTTTCATGGAATCCGT | 900 |  |
|  | *icaC* | F-ATGGGACGGATTCCATGAAAAAGA  R- TAATAAGCATTAATGTTCAATT | 1100 |  |
|  | *icaD* | F- ATGGTCAAGCCCAGACAGAG  R- AGTATTTTCAATGTTTAAAGCAA | 198 |  |
|  | *clfA* | CGC CGG TAA CTG GTG AAG CT  TGC TCT CAT TCT AGG CGC ACTT | 314 | (Soltani et al. 2019) |
|  | *clfB* | CCG GTA GTA AAT GCT GCT GTA  CAC TTT GAT TAG GGT CAA ATG TAG TC | 103 |  |
|  | *fnbA* | TGG TAC TGA TGA AGT TGA TTT TAG AAC  CAT TAT CCC AAG TTA AGG TAT ATC CTC | 101 |  |
