# Supplementary material for: Virulence determinants and toxin profile of methicillin resistant Staphylococcus aureus from commercial cheese in Bangladesh: A public health risk
Source: PLoS One. 2026 Jun 11;21(6):e0350222. doi: 10.1371/journal.pone.0350222 (PMC13257977; doi:10.1371/journal.pone.0350222)
Supplement: S2 Table — (DOCX) [file pone.0350222.s002.docx]

**Table S2: Antibiotics used in sensitivity test against *S. aureus* isolates**

| **Drug Name** | **Concentration**  **(µg)** | **Interpretive categories and Inhibition zone diameter (mm)** | | |
| --- | --- | --- | --- | --- |
|  |  | **R** | **I** | **S** |
| Penicillin G | 10 | ≤ 28 | - | ≥ 29 |
| Azithromycin | 15 | ≤ 13 | 14-17 | ≥ 18 |
| Trimethoprim-Sulfamethoxazole | 30 | ≤ 12 | 13-17 | ≥ 18 |
| Gentamycin | 10 | ≤ 12 | 13-14 | ≥ 15 |
| Ceftaroline | 30 | ≤ 19 | 20-24 | ≥ 25 |
| Cefoxitin | 30 | ≤ 24 | - | ≥ 25 |
| Chloramphenicol | 30 | ≤ 12 | 13-17 | ≥ 18 |
| Linezolid | 10 | ≤ 20 | - | ≥ 21 |
| Ciprofloxacin | 5 | ≤ 15 | 16-20 | ≥ 21 |
| Levofloxacin | 5 | ≤ 15 | 16-18 | ≥19 |
| Norfloxacin | 10 | ≤ 12 | 13-16 | ≥17 |
| Tetracycline | 30 | ≤ 14 | 15-18 | ≥ 19 |
| Doxycycline | 30 | ≤ 12 | 13-15 | ≥ 16 |
