# Supplementary material for: Virulence determinants and toxin profile of methicillin resistant Staphylococcus aureus from commercial cheese in Bangladesh: A public health risk
Source: PLoS One. 2026 Jun 11;21(6):e0350222. doi: 10.1371/journal.pone.0350222 (PMC13257977; doi:10.1371/journal.pone.0350222)
Supplement: S3 Table — (DOCX) [file pone.0350222.s003.docx]

**Table S3: Primer sets for detecting virulence and antimicrobial resistance genes**

|  | **Target genes** | **Nucleotide Sequences (5’-3’)** | **Amplicon size (bp)** | **References** |
| --- | --- | --- | --- | --- |
| Enterotoxin genes | *SEa* | F- GGTTATCAATGTGCGGGTGG  R CGGCACTTTTTTCTCTTCGG | 102 | (Mehrotra et al., 2000) |
|  | *SEb* | F- GTATGGTGGTGTAACTGAG  R- CCAAATAGTGACGAGTTAGG | 164 |  |
|  | *SEc* | F-AGATGAAGTAGTTGTGTATGG  R-CACACTTTTAGAATCAACCG | 451 |  |
|  | *SEd* | F-CGATTAGGAGAAAATAAAA  R-TCTCTCGTTCTCCGCTCAT | 278 |  |
|  | *SEe* | F- GGTTTTTTCACAGGTCATCC  R-CTTTTTTTTCTTCGGTCAATC | 209 |  |
| Exfoliative Toxin genes | *eta* | F- GCAGGTGTTGATTTAGCATT  R- AGATGTCCCTATTTTTGCTG | 93 | (Mehrotra et al., 2000) |
|  | *etb* | F- ACAAGCAAAAGAATACAGCG  R- GTTTTTGGCTGCTTCTCTTG | 226 |  |
| Toxic shock syndrome toxin gene 1 | *TSST-1* | F- ACCCCTGTTCCCTTATCATC  R- TTTTCAGTATTTGTAACGCC | 326 |  |
| Antibiotic-Resistant Genes  (Set 1) | *TEM* | F- GCG GAA CCC CTA TTC G  R- ACC AAT GCT TAA TCA GTG AG | 964 | (Olesen et al., 2004) |
|  | *CTX-M* | F- ATG TGC AGY ACC AGT AAR GTK ATG GC  R- TGG GTR AAR TAR GTS ACC AGA AYS AGC GG | 592 | (Munday et al., 2004) |
|  | *CTX-M-1* | F- GGT TAA AAA ATC ACT GCG TC  R- TTG GTG ACG ATT TTA GCC GC | 863 | (Eckert et al., 2004) |
|  | *CTX-M-2a* | F- GAT GAG ACC TTC CGT CTG GA  R- CAG AAA CCG TGG GTT ACG AT | 397 | (Dierikx et al., 2010) |
| Antibiotic-Resistant Genes  (Set 2) | *SHV* | F- TTA TCT CCC TGT TAG CCA CC  R- GAT TTG CTG ATT TCG CTC GG | 795 | (Dierikx et al., 2010) |
|  | *OXA-1* | F- GGC ACC AGA TTC AAC TTT CAA G  R- GAC CCC AAG TTT CCT GTA AGT G | 564 | (Ogutu et al., 2015) |
| Antibiotic-Resistant Genes  (Set 3) | *CMY* | F- GAC AGC CTC TTT CTC CAC A  R- TGG AAC GAA GGC TAC GTA | 1007 | (Zhao et al., 2003) |
|  | *NDM-1* | F- CTT CCA ACG GTT TGA TCG TC  R- TAG TGC TCA GTG TCG GCA TC | 465 | (Islam et al., 2013) |
