# Supplementary material for: Virulence determinants and toxin profile of methicillin resistant Staphylococcus aureus from commercial cheese in Bangladesh: A public health risk
Source: PLoS One. 2026 Jun 11;21(6):e0350222. doi: 10.1371/journal.pone.0350222 (PMC13257977; doi:10.1371/journal.pone.0350222)
Supplement: S4 Table — (DOCX) [file pone.0350222.s004.docx]

**Table S4: Distribution of *S. aureus*, MRSA, virulence and antibiotic resistant genes**

| **Type of genes** | **Name of genes** | **Number of positive isolates** | **Percentages (%)** |
| --- | --- | --- | --- |
| *S. aureus* | nuc | 78/120 | 65.00 |
| MRSA | mecA | 36/120 | 30.00 |
| Staphylococcal enterotoxins | *SEa* | 13/78 | 16.67 |
|  | *SEb* | 0/78 | 0.00 |
|  | *SEc* | 7/78 | 8.97 |
|  | *SEd* | 0/78 | 0.00 |
|  | *SEe* | 0/78 | 0.00 |
| Exfoliative toxins | *etA* | 0/78 | 0.00 |
|  | *etB* | 0/78 | 0.00 |
|  | *TSST-1* | 20/78 | 25.64 |
| Antibiotic resistant genes | *CTX-M-2a* | 69/78 | 88.46 |
|  | *CTX-M-1* | 0/78 | 0.00 |
|  | *CTX-M* | 0/78 | 0.00 |
|  | *OXA-1* | 0/78 | 0.00 |
|  | *TEM* | 20/78 | 25.64 |
|  | *CMY* | 0/78 | 0.00 |
|  | *SHV* | 0/78 | 0.00 |
|  | *NDM-1* | 0/78 | 0.00 |
