# Supplementary material for: Virulence determinants and toxin profile of methicillin resistant Staphylococcus aureus from commercial cheese in Bangladesh: A public health risk
Source: PLoS One. 2026 Jun 11;21(6):e0350222. doi: 10.1371/journal.pone.0350222 (PMC13257977; doi:10.1371/journal.pone.0350222)
Supplement: S5 Table — (DOCX) [file pone.0350222.s005.docx]

**Table S5: Phenotypic and genotypic characterization of biofilm production**

| **Type of genes** | **Name of genes** | **Number of positive isolates** | **Percentages (%)** |
| --- | --- | --- | --- |
| Congo Red (+ve) |  | 67/78 | 85.90 |
| Congo Red (-ve) |  | 11/78 | 14.10 |
| Biofilm (+ve) |  | 63/78 | 80.77 |
| Biofilm (-ve) |  | 15/78 | 19.23 |
| Strong biofilm producer |  | 26/78 | 33.33 |
| Moderate biofilm producer |  | 12/78 | 15.38 |
| Weak biofilm producer |  | 22/78 | 28.21 |
| Biofilm regulatory genes | *icaA* | 57/78 | 73.08 |
|  | *icaB* | 0/78 | 0.00 |
|  | *icaC* | 0/78 | 0.00 |
|  | *icaD* | 42/78 | 53.85 |
|  | *clfA* | 61/78 | 78.21 |
|  | *clfB* | 48/78 | 61.54 |
|  | *fnbA* | 54/78 | 69.23 |
