# Supplementary material for: Virulence determinants and toxin profile of methicillin resistant Staphylococcus aureus from commercial cheese in Bangladesh: A public health risk
Source: PLoS One. 2026 Jun 11;21(6):e0350222. doi: 10.1371/journal.pone.0350222 (PMC13257977; doi:10.1371/journal.pone.0350222)
Supplement: Attachment — Submitted filename: Response to Reviewer on 2nd review.docx [file pone.0350222.s016.docx]

**Response to Reviewer**

**“Virulence Determinants and Toxin Profile of Methicillin-Resistant *Staphylococcus aureus* from Commercial Cheese in Bangladesh: A Public Health Risk”**

Dear Editor

I would like to express my gratitude to you for your kind handling of our manuscript. First, I would like to thank the reviewers for their valuable comments and criticisms, which made our manuscript more scientific and pertinent. It took us a bit long time to submit our feedback because, according to our reviewer's comment, we performed some parts of the experiment again and tried to trace back so that we could resolve the shortcomings of our study.

I'm asking the reviewers to look at the detailed response for their insightful feedback, which is listed below.

Dear Reviewer 1:

First, we would like to express our gratitude for your time to review our manuscript and for providing scientific criticism on different aspects of our manuscript. May I request you to have your kind attention again on the replies we provided below against your specific comments.

Reviewer: 1

The authors of this paper characterize the Staphylococcus aureus isolates from cheese samples in Bangladesh, according to their antibiotic resistance patterns, enterotoxin gene profiles and their biofilm formation ability. The result of this study is interesting and should be of interest to the readership of your journal.

Thank you very much for your appreciative comments on our submitted manuscript.

Line 45 to Line 151: corrected.

Line 172: Why you have sampled only 1g from each sample? You must sample at least 10g.

Thank you for your attention to detail. Already corrected and the reply was submitted to the first reviewing process.

Line 176 and Line 177: Thank you for your attention to detail. Already corrected and the reply was submitted to the first reviewing process.

Line 177: you must precise the number of colonies chosen from each positive sample.

Thank you for your valuable suggestion. We have now clearly mentioned the number of colonies (2-3 colonies) from each culture-positive sample.

Line 186- Line 211: Already corrected in the first reviewing process.

Line 223: why you have used the PBS for the preparation of the bacterial suspension?

Thank you for your insightful comment. Phosphate-buffered saline (PBS) is used in cell culture for several key reasons, primarily to maintain osmotic balance and pH, ensuring cell viability and integrity during various procedures. It's an isotonic solution, meaning its salt concentration is similar to that of cells, preventing them from shrinking or bursting due to water movement.

Line 235: Corrected

Line 264: With the biochemical tests, you can not confirm the S. aureus species, as you have many Staphylococcus species which are coagulase positive. For this, you must perform other tests and confirm the obtained isolates with molecular method (PCR).

Thank you for your valuable observation. We concur that since there are other coagulase-positive *Staphylococcus* species, biochemical testing by alone is insufficient to confirm *Staphylococcus aureus*. Since the main focus of our investigation was molecular detection, PCR targeting the particular *nuc* gene was employed to confirm the presence of *S. aureus*.

Line 293 to Line 421: Corrected.

Line 424: you can compare your results with those obtained by TITOUCHE et al (2024). Investigation of Biofilm Formation Ability and Antibiotic Resistance of Staphylococcus aureus Isolates from Food Products.

Thank you for your suggestion. We added the comparison in our discussion part.

Line 455 to Line 504: Corrected.

Dear reviewer, we are grateful for your valuable comments and critiques on our manuscript. We believe your comments are logical to address and will fit the journal. We have tried to address all the valuable comments.

………………………………………………………………………………………………………

Dear Reviewer 3:

First, we want to extend our gratitude to you for your valuable comments. May I request you to have your kind attention again on the replies we provided below against your specific comments.

Reviewer: 3

The authors performed this study to determine the virulence and toxin profiles of methicillin-resistant *Staphylococcus aureus* isolated from commercial cheese in Bangladesh. This type of study is really important for public health. Unfortunately, this study has many serious drawbacks. I found many discrepancies in the study design, especially in antibiotic selection. I believe the authors should check the CLSI guidelines properly before selecting any antibiotics. Also, I believe the authors need to perform the study again.

We appreciate your insightful remarks and your acknowledgment of the significance of our research on the prevalence of Methicillin-Resistant *S. aureus* in commercial cheese in Bangladesh. We acknowledge the concerns regarding antibiotic selection and have taken your suggestions seriously. We have already reassessed our approach and conducted this part of the study following CLSI recommendations to address this issue. We think this has increased the accuracy of our investigation and reinforced our findings. We sincerely value your helpful criticism, which has enabled us to improve our work and guarantee its applicability to public health.

Regarding our publication, “Virulence Determinants and Toxin Profile of Methicillin-Resistant *Staphylococcus aureus* from Commercial Cheese in Bangladesh: A Public Health Risk”, we appreciate your thoughtful criticism and helpful suggestions. We have carefully reviewed your recommendations and made the necessary changes to improve the manuscript's quality, rigor, and clarity.

Below, we provide detailed responses to each point raised:

Line 120: How much distilled water? Did you make the distilled water sterile before using it?

Thank you for your critical evaluation. We have utilized 90 ml of distilled water, which was sterilized by autoclaving it properly at 121 °C and 15 PSI pressure. We have revised our writing regarding this.

Line 145: Reference for boiling method?

We have updated the reference section.

Tables 2 and 5: I recommend using these tables as supplementary material.

I appreciate your recommendation on Tables 2 and 5. We concur that both tables, which provide information on thermal cycling conditions, could be better suited as supplemental content to help the main text flow. Following this, we have updated the manuscript and added them as supplemental tables.

Line 162: “2.6 Multiplex PCR for detection of virulence factor genes” – but you also mentioned resistance genes here. Please modify the name of the section.
Also, did you check resistance genes before determining their phenotypic resistance profiles? Please clarify this.

Thank you for pointing this out. We have updated the section heading. Regarding your second point, we performed a phenotypic resistance profile before detecting resistance genes via PCR. We apologize for the confusion and have clarified this sequence in the revised manuscript.

Line 176-195:

- So, you used oxacillin for S. aureus, right? How did you interpret the data based on CLSI 2020? You have to check their MIC; you can’t come to an outcome for oxacillin using the disk diffusion test. CLSI 2020 clearly mentions that oxacillin disk testing is not reliable for S. aureus. I believe this is a serious drawback of your study. You should go through your study again.

- Moreover, how did you interpret the resistance profiles of ampicillin and amoxicillin? As far as I know, you can only use penicillin (10 units) for S. aureus, which is another drawback.

- The same is true for streptomycin, cefotaxime, cefuroxime, and nalidixic acid. You should check the CLSI 2020 guidelines before using any antibiotics.

I appreciate your insightful comments. Your thorough analysis and insightful remarks about the antibiotic susceptibility testing are greatly appreciated.

In compliance with the CLSI 2020 recommendations, I have carefully re-evaluated the antibiotic selection and interpretation criteria after going over the pertinent portion of my study again. In light of this, I updated the antibiotic panel to include 13 agents that are suitable and advised for *Staphylococcus aureus* in accordance with CLSI guidelines. In particular:

-Following CLSI 2020, I have excluded oxacillin disc diffusion findings, recognising that MIC testing is the proper technique for interpreting oxacillin in *S. aureus*.

-Additionally, I have reinterpreted ampicillin and amoxicillin and now only use penicillin (10 units) for *S. aureus* β-lactam susceptibility profiling.

-Similarly, I have removed the antibiotics that CLSI does not advise for *S. aureus* susceptibility testing, including streptomycin, cefotaxime, cefuroxime, and nalidixic acid.

These corrections have been incorporated into the revised dataset and analysis, ensuring full alignment with the CLSI 2020 standards. I believe these updates address the concerns raised and improve the overall reliability of the study.

Line 189- Line 214: Updated and Corrected.

Line 246: You can’t determine MRSA by using only the mecA gene. There are several resistance genes that can also be responsible for MRSA, e.g., mecB, mecC, mecD, or others. This is another drawback of this study.

Regarding MRSA detection, I appreciate your insightful assessment. As you correctly point out, although the *mecA* gene is most frequently linked to methicillin resistance in Staphylococcus aureus, other *mec* homologs, including *mecB, mecC*, and *mecD*, have also been found to be involved in the MRSA phenotype.

Since *mecA* is still the most common and extensively researched marker for MRSA identification, especially in clinical and epidemiological settings, we concentrated on it in this investigation. I do admit, though, that *mecA* alone might not be able to identify all possible MRSA strains, particularly uncommon or newly discovered varieties. In order to present a more impartial analysis of the findings, I will address this significant constraint in the discussion section.

Dear reviewer, we are grateful for your valuable comments and critiques on our manuscript. We believe your comments are logical to address and will fit the journal. We have tried to address all the valuable comments.

Thanks again, and have a good day.

Best regards

Prof. Dr. Ferdaus Mohd Altaf HOSSAIN
