# Supplementary material for: Virulence determinants and toxin profile of methicillin resistant Staphylococcus aureus from commercial cheese in Bangladesh: A public health risk
Source: PLoS One. 2026 Jun 11;21(6):e0350222. doi: 10.1371/journal.pone.0350222 (PMC13257977; doi:10.1371/journal.pone.0350222)
Supplement: Attachment — Submitted filename: plos one.docx [file pone.0350222.s015.docx]

Line 95: write the abbreviated form of International Institute for Diarrheal Disease Research within bracket

Line 132: change the heading 2.3 (Microbiological evaluation of Cheese sample). Aren’t the isolation, identification, molecular detection and antibiotic sensitivity test included in microbiological evaluation? Your writing resembles that only CFU counting represents the microbiological evaluation. You can change the heading as ‘’ total count of Staphylococcus genus’’.

Line 163: What are the modifications?

Line 213: Recheck this heading and make it shorter

Line 228: This study performed in Bangladesh. That’s why it is ok to compare the findings with the recommended limit of BFSA. But why the findings are compared to that of Standards Authority of India, and the European Union bypassing other countries like USA, Australia and others?

Line 228-229: Reference?

Line 286-287: 3.5 Toxin, Virulence, and antibiotic-resistant genes profile of Staphylococcus aureus isolated from cheese: What about SEb and SEd. Clarify their percentage.

Figure 6: it will be easier to understand if you represent the two pictures of PCR with two different figure names

Figure 8: same as figure 6

Figure 9: Try to increase the sharpness and clarity of the given diagram.

Line 341: Why is a matter of concern?

Discussion: line 388-394: Recheck the sequence and organization of the provided information. At first you wrote about the percentage of S. aureus and compared it with the existing data, then wrote about MRSA in the same manner. After that you repeated the S. aureus again. Try to write in more organized way.

Line 437: is it TEM or blaTEM?

**Overall evaluation:** Recheck the whole manuscript again for grammatical errors. The organism’s name and genes name must be in italic form. The headings under different section should be rechecked to make it simpler, smarter and shorter.
