# Supplementary material for: Virulence determinants and toxin profile of methicillin resistant Staphylococcus aureus from commercial cheese in Bangladesh: A public health risk
Source: PLoS One. 2026 Jun 11;21(6):e0350222. doi: 10.1371/journal.pone.0350222 (PMC13257977; doi:10.1371/journal.pone.0350222)
Supplement: Attachment — Submitted filename: Response to Reviewers on latest review.docx [file pone.0350222.s017.docx]

**Response to Reviewer**

**PONE-D-24-38694R2**

**“Virulence Determinants and Toxin Profile of Methicillin-Resistant *Staphylococcus aureus* from Commercial Cheese in Bangladesh: A Public Health Risk”**

Dear Editor

I sincerely appreciate your kind handling of our manuscript. I am also grateful to the reviewers for their insightful comments and constructive criticisms, which have significantly improved the scientific rigor and relevance of our work. The revision process required additional time, as we repeated certain experiments and carefully re-examined aspects of the study in order to address the reviewers’ concerns and resolve identified limitations.

I'm asking the reviewers to look at the detailed response for their insightful feedback, which is listed below.

Dear Reviewer 4:

First, we would like to express our gratitude for your time to review our manuscript and for providing scientific criticism on different aspects of our manuscript. May I request you to have your kind attention again on the replies we provided below against your specific comments.

Reviewer: 4

Abstract:

Line 28-30: In my opinion, the authors could represent this portion in another way. Suddenly started about MRSA in the very next sentence. The relationship of MRSA and cheese might come first and then objective of the research could be mentioned.

Thank you for your constructive suggestion. We have revised Lines 28–30 to first establish the relationship between MRSA and cheese, followed by a clear statement of the study objective. The flow of the text has been improved accordingly.

Line 35: Please rephrase this sentence.

Thank you for your comment. The sentence in Line 35 has been rephrased for improved clarity and readability.

Line 36: please write as……….. polymerase chain reaction (PCR)

Thank you for your suggestion. The abbreviation has now been correctly written as polymerase chain reaction (PCR) in Line 36.

Line 41-42: Please mention the safety threshold briefly.

Thank you for your valuable comment. The relevant safety threshold has now been briefly mentioned in Lines 41–42.

Introduction:

Authors did not establish link between cheese and MRSA.

No research gap was mentioned.

Previous research on MRSA and cheese on global or national contexts was not mentioned.

Sources of contamination with MRSA should be discussed clearly.

Thank you for these insightful comments. The Introduction section has been substantially revised to:

- Clearly establish the link between cheese and MRSA,
- Highlight the existing research gap,
- Incorporate relevant global and national studies on MRSA in cheese, and
- Clearly discuss possible sources of MRSA contamination.

Line 57-58: Please give reference.

Appropriate references have now been added to support the statement in Lines 57–58.

Line 60-61: need reference(s).

Relevant references have been added to Lines 60–61.

Line 67-68: need reference(s)

Supporting references have now been included for the statement in Lines 67–68.

Line 69-70: need reference(s).

Necessary references have been added to Lines 69–70.

Line 57-68: Need multiple references for each statement.

Thank you for your valuable observation. Multiple appropriate references have now been added for each statement between Lines 57–68 to strengthen the scientific support.

Materials and Methods:

Line `112-113: Only one raw milk cheese included? Why? Making comparison was not difficult?

Thank you for your thoughtful comment. In the study area, only one type of cheese produced from raw milk was available during the sampling period. Therefore, inclusion of additional raw milk cheese types and comparative analysis was not feasible.

Line 140-141: mention the rationale of using pour plate method.

Thank you for your comment. We apologize for the confusion. The streaking method was used instead of the pour plate method.

Line 148-149: Why so many references for boiling method? Single reference was not enough? Why boiling method? Why did not any Kit?

Thank you for raising this important point. The number of references has been reduced to the most relevant one. The boiling method was chosen due to its cost-effectiveness, simplicity, and suitability for resource-limited laboratory settings without compromising PCR performance. However, we performed the extraction of genomic DNA using kit again.

Line 175: why CLSI, 2020? Why not recent?

Thank you for your comment. We apologize for the oversight. Antimicrobial susceptibility testing (AST) was performed following the most recent CLSI 2025 guidelines, and this has now been corrected and clearly stated in Line 175.

Line 194-203: Use references.

Appropriate and relevant references have now been added to support the methodology described in Lines 194–203.

Results:

As these findings were reviewed by previous reviewers, I don’t have queries.

We sincerely thank the reviewer for reviewing the Results section and for having no further queries.

Discussion:

Why 100%? Most of the authors found lower percentage of Staph. Aureus in their researches. The authors mentioned country and production procedure variations. But the commercial cheese production of renowned Brands follows the International standards, even in Bangladesh. In my opinion, authors should establish scientific basis of this finding.

Thank you for this important and insightful comment. The Discussion section has been thoroughly revised to establish a clear scientific basis for the detection rate of *S. aureus*. We have now incorporated detailed explanations and relevant supporting literature has also been added to strengthen this interpretation.

As all samples exceeded the EU recommended standard, then what could be the authors decision about the commercial cheese in Bangladesh?

Thank you for this valuable observation. We have now clearly stated the authors’ interpretation and decision regarding the microbiological safety of commercial cheese in Bangladesh in the revised Discussion section, emphasizing public health implications and the need for stricter monitoring and enforcement.

Conclusion:

Conclusion became too long. Should be more precise and shorter.

Thank you for your suggestion. The Conclusion section has now been shortened and refined to present only the key findings and implications in a more concise and focused manner.

Line 427: After finishing the whole experiments, showing all findings, why did authors mention “may be’?

Thank you for pointing this out. The use of the term “may be” was unintentional. It has now been removed, and the sentence has been revised to reflect definitive findings based on the experimental results.

………………………………………………………………………………………………………

Dear Reviewer 5:

First, we want to extend our gratitude to you for your valuable comments. May I request you to have your kind attention again on the replies we provided below against your specific comments.

Reviewer: 5

The manuscript entitled “Virulence Determinants and Toxin profile of Methicillin Resistant Staphylococcus aureus from Commercial Cheese in Bangladesh: A public Health Risk” presented the virulence and toxin factors of MRSA from commercial cheese in Bangladesh. The Authors also showed the AMR profile of MRSA by both phenotypic and genotypic approach. The manuscript has great importance in the field of AMR and food safety. However, the manuscript requires some corrections. There are areas of improvement. The knowledge gap should include why the study is different from other studies in Bangladesh. The discussion section has some repetitions. Please check the whole manuscript for English corrections. There is a serious issue in the methodology of bacterial load determination.

We sincerely thank the reviewer for acknowledging the significance of our study on MRSA in commercial cheese in Bangladesh and for the constructive comments. The knowledge gap in the Introduction has been revised to clearly highlight how this study differs from previous research, integrating virulence determinants, toxin profiling, and antimicrobial resistance analysis using both phenotypic and genotypic approaches. The Discussion has been reorganized to remove repetitions and improve clarity, and the bacterial load methodology has been reassessed, clarified, and supported with appropriate references. The manuscript has also been thoroughly edited for English language and readability.

We greatly appreciate your feedback, which has strengthened the manuscript and enhanced its relevance to antimicrobial resistance and food safety.

Below, we provide detailed responses to each point raised:

Title: Why the word “public” starts with the small “p”.

Thank you for pointing this out. The title has been revised to capitalize “Public” appropriately.

Line no. 42: Please add “and” before “Food Safety and Standards Authority India (FSSAI)”.

Thank you for your observation. “and” has been added for correct grammar in Line 42.

Line no. 46: showed a moderate level of resistance. Check the sentence.

Thank you. The antibiotic sensitivity part has been revised for clarity.

The gene name should be Italic. Check the uniformity of gene and species names throughout the manuscript.

Thank you for this important comment. All gene names and species names have been italicized consistently throughout the manuscript.

Please check the whole manuscript for similar minor errors to improve the quality of the manuscript.

We appreciate your suggestion. The entire manuscript has been thoroughly reviewed and corrected for minor grammatical, formatting, and typographical errors.

Line no. 110: Are all the brands from Bangladesh or imported as well?

Thank you for this query. All sampled brands were locally produced in Bangladesh.

Line 134-137: As per standard, the sample amount should be 10 grams. Thereafter, for ten-fold dilution, you must have to maintain 1:10 that is one part sample and 9 parts diluent. However, the Authors have not maintained this ratio. They used one gram sample and 900 microliter (0.9 ml) diluent that is 1: 0.9 ratio. your total volume is approximately 1.9mL. Your dilution factor is 1/1.9, which is approximately 10⁻⁰·²⁸, not 10⁻¹.

It is a serious mistake in the sample preparation and it will misinterpret the findings.

Thank you for highlighting this important issue. We acknowledge the discrepancy. The dilution factor and sample preparation have been revised and corrected following standard protocols (10 g sample with 1:10 dilutions). The methodology section now reflects this correction, and the calculations have been updated accordingly.

Line no. 150-151: Table 01, contains primers of nuc, mecA, and toxin genes.

The supplementary table has been properly referenced and included.

Line no. 156: Table 01. Where is the supplementary table? After Table 1, where is Table 02?

The supplementary table has been properly referenced and included.

Line no. 162-163: The sentence is not clear. Please mention the name of the test with the interpretation criteria.

This part has been revised and written correctly.

Line no. 169-170: Approximate concentration of 1.5*10-8 CFU ml-1.

Corrected.

Line no. 247: total samples.

Corrected.

Line no. 380-382: Please mention the causes of differences in occurrences of MRSA in cheese particularly for Bangladesh.

Thank you. The Discussion has been revised to explain the differences in MRSA occurrence, considering factors such as variations in hygiene practices, production methods, storage, and regulatory implementation in Bangladesh.

………………………………………………………………………………………………………

Dear Reviewer 6:

We sincerely appreciate the time and effort you have devoted to reviewing our manuscript and providing valuable scientific feedback. We kindly request your attention to the responses we have prepared below, addressing each of your specific comments.

Reviewer 6:

Line 95: write the abbreviated form of International Institute for Diarrheal Disease Research within bracket

Thank you for your suggestion. The whole introduction part has been revised to addressed the amendments for better clarity and scientific quality.

Line 132: change the heading 2.3 (Microbiological evaluation of Cheese sample). Aren’t the isolation, identification, molecular detection and antibiotic sensitivity test included in microbiological evaluation? Your writing resembles that only CFU counting represents the microbiological evaluation. You can change the heading as ‘’ total count of Staphylococcus genus’’.

Thank you for pointing this out. The methodology part has been revised regarding the heading and overall workflows for the suitability of the manuscript for the journal.

Line 163: What are the modifications?

Thank you for the query. All modifications made to the methodology have now been explicitly described for clarity.

Line 213: Recheck this heading and make it shorter

All the comments regarding the headings has been addressed.

Line 228: This study performed in Bangladesh. That’s why it is ok to compare the findings with the recommended limit of BFSA. But why the findings are compared to that of Standards Authority of India, and the European Union bypassing other countries like USA, Australia and others?

Thank you for your comment. We compared our findings with FSSAI and EU standards to provide a perspective on the food safety level in Bangladesh relative to widely recognized international benchmarks. These standards are also commonly referenced and followed in our country, making them relevant for contextual comparison.

Line 228-229: Reference?

All relevant references were cited where needed.

Line 286-287: 3.5 Toxin, Virulence, and antibiotic-resistant genes profile of *Staphylococcus aureus* isolated from cheese: What about *SEb* and *SEd*. Clarify their percentage.

Thank you. No isolates were positive for *SEb* and *SEd.*

Figure 6: it will be easier to understand if you represent the two pictures of PCR with two different figure names.

Corrected.

Figure 8: same as figure 6

Corrected.

Figure 9: Try to increase the sharpness and clarity of the given diagram.

Thank you. All the diagrams given with the manuscript were prepared with high clarity and sharpness again.

Line 341: Why is a matter of concern?

Addressed properly.

Discussion: line 388-394: Recheck the sequence and organization of the provided information. At first you wrote about the percentage of S. aureus and compared it with the existing data, then wrote about MRSA in the same manner. After that you repeated the S. aureus again. Try to write in more organized way.

Thank you for this observation. The Discussion section has been reorganized to first present the findings for S. aureus, followed by MRSA, avoiding repetition and improving logical flow.

Line 437: is it TEM or blaTEM?

Thank you for pointing this out. The gene name has been corrected to *blaTEM.*

Overall evaluation: Recheck the whole manuscript again for grammatical errors. The organism’s name and genes name must be in italic form. The headings under different section should be rechecked to make it simpler, smarter and shorter.

We appreciate your suggestion. The entire manuscript has been thoroughly revised for grammar, clarity, and readability. All organism and gene names are now in italic form, and headings have been simplified and shortened for better presentation.

………………………………………………………………………………………………………

Dear Reviewer 7:

We sincerely appreciate your time and valuable feedback and invite you to review our responses to each of your specific comments.

Reviewer 7:

I have reviewed the manuscript "Virulence Determinants and Toxin Profile of Methicillin-Resistant Staphylococcus aureus from Commercial Cheese in Bangladesh: A Public Health Risk" for the first time. The authors have justified or corrected the observations made by reviewers in previous rounds. However, I believe there are still several errors or clarifications that need to be made, particularly in the methods and results.

We sincerely thank the reviewer for taking the time to evaluate our manuscript and for acknowledging the revisions made in response to previous rounds of review. We appreciate your constructive feedback and have carefully addressed all additional comments and clarifications, particularly in the Methods and Results sections, to improve the accuracy, clarity, and overall quality of the manuscript.

Material and Methods

Sections 2.4, 2.5, 2.6 and 2.7: Authors must include and mention the use of positive and negative controls.

Thank you for this important observation. At the time of the experiments, we were unable to include a positive control due to the unavailability of an appropriate reference strain in our laboratory. However, negative controls were consistently used and are now clearly stated in the revised manuscript.

Table 1: The primers listed are not compatible with those used in the references indicated. Particularly in refs. 16 and 17, the primers are completely different. In the other cases (ref. 18), there are differences in some nucleotides. Authors should verify each primer in Tables 1, 3, and 4.

Thank you for pointing this out. All primers listed in Tables 1, 3, and 4 have been carefully rechecked and verified against the corresponding original references.

Section 2.4/Line 148: It is excessive to use 5 references to cite the boiling method. Reduce to 1 or a maximum of 2.

The number of references citing the boiling method has been reduced to the most relevant one.

Section 2.6/Line 167: Authors must appropriately cite the CLSI manual used and not any other indirect reference.

Thank you for highlighting this issue. The CLSI manual has now been appropriately cited.

Sections 2.4 + 2.7: I recommend merging these two sections, as they are all PCR-based. Also, merge tables (1, 3, and 4)—note that Table 2 does not exist—and maintain the separation of primer groups by category as they were.

We appreciate this recommendation. This part has been revised.

These analyses are inappropriate and should be withdrawn. The fragment considered (233 bp) is very small and highly conserved in S. aureus (I blasted the OR096215 sequence and found nearly 2,000 sequences with 100% identity). The resolution of the phylogenetic tree does not allow for appropriate inferences between identical sequences. Any discussion or conclusion based on this analysis must be withdrawn.

Thank you for this critical assessment. I addressed the amendments that you suggested.

Results:

Since all PCR assays are from references and were not developed in the present study, I consider the gel figures (Figs. 3, 4, 6, 7, 8, 10B) unnecessary in the main body of the manuscript. I suggest transferring them as supplementary material.

Thank you for this suggestion. Checked.

Figure 3: The authors indicate an expected amplification size of 280 bp for the nuc gene, but the figure shows bands above 300 bp. What is the reason for this?

Thank you for noting this discrepancy. This issue has now been addressed and clarified in the revised Results section and figure legend.

Figure 4: In the legend, the authors indicate that the samples in lanes 5 and 6 are negative. However, the same positive band can be seen in lanes 1 through 4.

Thank you for pointing this out. The legend and interpretation of Figure 4 have been revised for accuracy and clarity.

3.4: The authors refer to Table 4, which currently lists primers against biofilm-producing genes. Verify.

Thank you. This section has been revised.

Figure 8: In panels A and B, the authors indicate other sizes such as "100 bp." Since the 100 band is not visible, I suggest only identifying the 500 band, which is the most intense. In panel B, something similar occurs as in Figure 3. The authors indicate that the detection band is 397 bp, however, in the gel photo, it appears to be below 300 bp. Is it possible that electrophoresis generated a curve? In any case, the use of positive controls is necessary to validate these results.

Thank you for this detailed observation. Figure annotations have been simplified as suggested, inconsistencies in band size interpretation have been addressed, and the role of positive controls has been clarified in the revised text.

Finally, I suggest a review of the language to avoid overly colloquial idioms and phrases.

Thank you for the suggestion. The entire manuscript has been thoroughly revised for language, tone, and scientific clarity to remove colloquial expressions.

………………………………………………………………………………………………………

Dear Reviewer 8:

We appreciate your time and valuable feedback on our manuscript.

Reviewer 8:

The article is interesting and congratulations for your work. The authors did almost all required revisions.

Thank you very much for your kind words and positive evaluation of our work. We sincerely appreciate your time and effort in reviewing the manuscript and are grateful for your constructive feedback throughout the review process.

Dear reviewers, we are grateful for your valuable comments and critiques on our manuscript. We believe your comments are logical to address and will fit the journal. We have tried to address all the valuable comments.

Thanks again, and have a good day.

Best regards

Prof. Dr. Ferdaus Mohd Altaf HOSSAIN
