# Supplementary material for: Virulence determinants and toxin profile of methicillin resistant Staphylococcus aureus from commercial cheese in Bangladesh: A public health risk
Source: PLoS One. 2026 Jun 11;21(6):e0350222. doi: 10.1371/journal.pone.0350222 (PMC13257977; doi:10.1371/journal.pone.0350222)
Supplement: Attachment — Submitted filename: Response to Reviewers.docx [file pone.0350222.s018.docx]

**Response to Reviewer**

**PONE-D-24-38694R2**

**“Virulence Determinants and Toxin Profile of Methicillin-Resistant *Staphylococcus aureus* from Commercial Cheese in Bangladesh: A Public Health Risk”**

Dear Editor

I sincerely appreciate your thoughtful handling of our manuscript. I am also grateful to the reviewers for their valuable insights and constructive feedback, which have greatly strengthened the scientific quality and relevance of our study.

We kindly invite the reviewers to consider our detailed, point-by-point responses to their comments, provided below.

Dear Reviewer 7:

First, we would like to express our gratitude for your time to review our manuscript and for providing scientific criticism on different aspects of our manuscript. May I request you to have your kind attention again on the replies we provided below against your specific comments.

Reviewer: 7

The authors have appropriately corrected or justified almost all of the observations made in the preliminary round of my reviews.

However, they persist in maintaining the phylogenetic analysis and its discussions, which are inappropriate, as already argued in the previous round: "These analyses are inappropriate and should be withdrawn. The fragment considered (233 bp) is very small and highly conserved in S. aureus (I blasted the OR096215 sequence and found nearly 2,000 sequences with 100% identity). The resolution of the phylogenetic tree does not allow for appropriate inferences between identical sequences. Any discussion or conclusion based on this analysis must be withdrawn." Withdrawing this analysis does not detract from the merit of the study. On the contrary, persisting on this point undermines its scientific soundness. I insist on completely withdrawing this analysis (methods, results, and discussions) to avoid inappropriate or unjustified inferences.

We sincerely thank the reviewer for their careful evaluation and for reiterating this important concern. We fully acknowledge the limitations associated with the phylogenetic analysis based on the short (233 bp) and highly conserved fragment, and we appreciate the reviewer’s clarification regarding the lack of discriminatory power and the risk of drawing unsupported inferences.

In response, we have completely removed all components related to the phylogenetic analysis from the revised manuscript, including the corresponding methods, results, figures, and discussion.

………………………………………………………………………………………………………

Dear Reviewer 5:

First, we want to extend our gratitude to you for your valuable comments. May I request you to have your kind attention again on the replies we provided below against your specific comments.

Reviewer: 9

This manuscript addresses a worthwhile public-health question and has potential value as a regional surveillance study on S. aureus/MRSA in commercial cheese.

However, in its current form, the paper has substantial methodological and reporting weaknesses that limit confidence in the quantitative conclusions. My comments as follows:

We sincerely thank the reviewer for recognizing the public health relevance and regional value of our study. We appreciate the constructive feedback regarding the methodological and reporting limitations. In response, we have carefully revised the manuscript to address these concerns, improving the clarity, methodological transparency, and overall robustness of our analyses. We hope the revisions have strengthened confidence in our findings.

Below, we provide detailed responses to each point raised:

1. Unclear unit of analysis / isolate count

The manuscript does not clearly explain how many isolates were actually analyzed. It mentions 72 cheese samples, but many results use denominators that suggest only 12 isolates. It is unclear whether the study used one isolate per sample, per brand, or per positive culture, which makes prevalence and resistance/toxin frequencies hard to interpret.

Thank you for highlighting this important point regarding the clarity of the unit of analysis. In the revised manuscript, we have clearly stated that a total of 120 cheese samples were collected over a six-month period from 12 commercially available brands. All subsequent analyses and interpretations were conducted based on these samples. We have revised the relevant sections to ensure consistency and to avoid any ambiguity regarding isolate numbers and denominators used throughout the manuscript.

2. Internal inconsistencies in results

The reported findings conflict across sections. Antibiotic resistance profiles differ between the abstract and Results, and the reported frequencies of biofilm and toxin genes are inconsistent. These are major result discrepancies, not minor wording issues.

Thank you for carefully identifying these inconsistencies. We acknowledge that the discrepancies in antibiotic resistance profiles, as well as the reported frequencies of biofilm formation and toxin genes, may have caused confusion. In the revised manuscript, all such inconsistencies have been thoroughly checked and corrected. The data have been harmonized across the Abstract, Results, and relevant sections to ensure accuracy, consistency, and clarity.

3. Problems in microbiological methods

The described pre-enrichment in nutrient broth may be acceptable for detecting presence/absence, but not for quantitative estimation of bacterial load. Since the study also reports CFU/g and compares counts to safety thresholds, the workflow appears methodologically inconsistent. The dilution scheme described is also nonstandard and unclear.

Thank you the for this important observation regarding the microbiological methodology. We apologize for the lack of clarity in the previous version. In the revised manuscript, we have clearly explained that each sample was divided into two portions: one portion was used for the detection and isolation of S. aureus and MRSA following enrichment procedures, while the other portion was processed separately for direct microbial enumeration to determine CFU/g without enrichment. We have also revised the description of the dilution and plating procedures to ensure clarity and alignment with standard quantitative methods.

4. Insufficiently robust identification strategy

The identification approach (MSA, Gram stain, catalase/coagulase, nuc PCR) is reasonable for confirming S. aureus, but the manuscript does not clearly explain isolate selection or controls. Given the unusually high claim of 100% S. aureus positivity in all cheese samples, stronger methodological detail is needed.

We appreciate this important comment. We have carefully revised the manuscript to improve the clarity and detail of the identification strategy. We would also like to clarify that the prevalence of S. aureus is not 100% as previously implied; the corrected results show a prevalence of 65%, while MRSA was detected in 30% of the samples. These values have been consistently updated throughout the manuscript. We have thoroughly checked and revised the relevant sections to ensure accuracy, clarity, and consistency.

5. Weak justification for resistance-gene panel

Several screened resistance genes, especially ESBL/carbapenemase genes like CTX-M, CMY, SHV, and NDM-1, are more typical of Gram-negative bacteria and are not well justified in an S. aureus study. Their biological relevance and interpretation are not adequately discussed, and sequencing confirmation is lacking.

Thank you for highlighting this important point. We acknowledge that ESBL and carbapenemase genes such as CTX-M, CMY, SHV, and NDM-1 are primarily associated with Gram-negative organisms, and their biological relevance in S. aureus is not well established. In the revised manuscript, we have carefully reconsidered this aspect and clarified the interpretation of these findings. We have avoided overstatement and emphasized that, if detected, such genes may reflect potential horizontal gene transfer events or methodological limitations, rather than established resistance mechanisms in S. aureus. The discussion has been revised accordingly to ensure scientific accuracy and caution in interpretation.

6. Overstated toxin conclusions

The study reports toxin genes by PCR, but the manuscript phrases this as if actual toxins were present in the cheese. Gene detection only shows the potential for toxin production, not confirmed toxin expression or toxin presence in the food.

We thank the reviewer for this important clarification. We fully agree that the detection of toxin genes by PCR indicates only the genetic potential for toxin production and does not confirm actual toxin expression or presence in the cheese. We would like to clarify that we did not intend to imply confirmed toxin production. In the revised manuscript, we have carefully reviewed and refined the wording throughout to ensure that all statements accurately reflect gene detection rather than toxin presence, thereby avoiding any potential overinterpretation.

7. Weak statistical analysis

The statistical section lacks a strong analytical framework. Comparing study findings directly to regulatory limits using p-values is not appropriate, and the paper does not provide confidence intervals, clear hypotheses, between-group comparisons, or detailed data presentation.

Thank you for highlighting this important issue. We acknowledge that the previous version lacked sufficient analytical depth and clarity. In the revised manuscript, we have strengthened the statistical framework by clearly defining the analytical approach, removing inappropriate comparisons with regulatory limits using p-values, and improving overall data presentation.

8. Inadequate phylogenetic analysis

The phylogenetic methods are not described in enough detail to support the claim of identifying a “new strain.” Key details are missing, including number of sequences, gene region, quality control, accession numbers, and reference selection.

We thank the reviewer for this important observation. We acknowledge that the phylogenetic analysis was not described with sufficient detail to support such claims. In response, and in line with the reviewer’s concerns, we have completely removed all components related to the phylogenetic analysis from the revised manuscript, including the associated methods, results, and discussion. This revision ensures greater scientific rigor and avoids unsupported interpretations.

Dear Reviewers,

We sincerely thank you for your valuable comments and constructive critiques on our manuscript. We greatly appreciate the time and expertise you have invested in evaluating our work. We found your suggestions insightful and highly relevant, and we believe they have significantly improved the quality and suitability of the manuscript for the journal. We have carefully addressed all comments and revised the manuscript accordingly.

Thanks again, and have a good day.

Best regards

Prof. Dr. Ferdaus Mohd Altaf HOSSAIN
