# Supplementary material for: Virulence determinants and toxin profile of methicillin resistant Staphylococcus aureus from commercial cheese in Bangladesh: A public health risk
Source: PLoS One. 2026 Jun 11;21(6):e0350222. doi: 10.1371/journal.pone.0350222 (PMC13257977; doi:10.1371/journal.pone.0350222)
Supplement: Peer review history

# Peer review: Decision Letter 0

**DOI:** 10.1371/journal.pone.0350222.r001

**Authors:** Reham Mokhtar ELTarabili

20 Sep 2024

PONE-D-24-38694

Molecular detection of super-antigenic Methicillin-Resistant Staphylococcus aureus from commercial cheese in Bangladesh

PLOS ONE

Dear Dr. Hossain,

Thank you for submitting your manuscript to PLOS ONE. After careful consideration, we feel that it has merit but does not fully meet PLOS ONE’s publication criteria as it currently stands. Therefore, we invite you to submit a revised version of the manuscript that addresses the points raised during the review process.

==============================

I have completed my evaluation of your manuscript. The reviewers recommend reconsideration of your manuscript following major revision. I invite you to resubmit your manuscript after addressing the comments below. Please resubmit your revised manuscript by Nov 04 2024 11:59PM.

When revising your manuscript, please consider all issues mentioned in the reviewers' comments carefully: please outline every change made in response to their comments and provide suitable rebuttals for any comments not addressed. Please note that your revised submission may need to be re-reviewed.

==============================

Please submit your revised manuscript by Nov 04 2024 11:59PM. If you will need more time than this to complete your revisions, please reply to this message or contact the journal office at plosone@plos.org. When you're ready to submit your revision, log on to https://www.editorialmanager.com/pone/ and select the 'Submissions Needing Revision' folder to locate your manuscript file.

Please include the following items when submitting your revised manuscript:

- A rebuttal letter that responds to each point raised by the academic editor and reviewer(s). You should upload this letter as a separate file labeled 'Response to Reviewers'.
- A marked-up copy of your manuscript that highlights changes made to the original version. You should upload this as a separate file labeled 'Revised Manuscript with Track Changes'.
- An unmarked version of your revised paper without tracked changes. You should upload this as a separate file labeled 'Manuscript'.

If you would like to make changes to your financial disclosure, please include your updated statement in your cover letter. Guidelines for resubmitting your figure files are available below the reviewer comments at the end of this letter.

If applicable, we recommend that you deposit your laboratory protocols in protocols.io to enhance the reproducibility of your results. Protocols.io assigns your protocol its own identifier (DOI) so that it can be cited independently in the future. For instructions see: https://journals.plos.org/plosone/s/submission-guidelines#loc-laboratory-protocols. Additionally, PLOS ONE offers an option for publishing peer-reviewed Lab Protocol articles, which describe protocols hosted on protocols.io. Read more information on sharing protocols at https://plos.org/protocols?utm_medium=editorial-email&utm_source=authorletters&utm_campaign=protocols.

We look forward to receiving your revised manuscript.

Kind regards,

Reham Mokhtar ELTarabili

Academic Editor

PLOS ONE

Journal Requirements:

When submitting your revision, we need you to address these additional requirements.

1. Please ensure that your manuscript meets PLOS ONE's style requirements, including those for file naming. The PLOS ONE style templates can be found at

https://journals.plos.org/plosone/s/file?id=wjVg/PLOSOne_formatting_sample_main_body.pdf and

https://journals.plos.org/plosone/s/file?id=ba62/PLOSOne_formatting_sample_title_authors_affiliations.pdf

2. PLOS ONE now requires that authors provide the original uncropped and unadjusted images underlying all blot or gel results reported in a submission’s figures or Supporting Information files. This policy and the journal’s other requirements for blot/gel reporting and figure preparation are described in detail at https://journals.plos.org/plosone/s/figures#loc-blot-and-gel-reporting-requirements and https://journals.plos.org/plosone/s/figures#loc-preparing-figures-from-image-files. When you submit your revised manuscript, please ensure that your figures adhere fully to these guidelines and provide the original underlying images for all blot or gel data reported in your submission. See the following link for instructions on providing the original image data: https://journals.plos.org/plosone/s/figures#loc-original-images-for-blots-and-gels.

In your cover letter, please note whether your blot/gel image data are in Supporting Information or posted at a public data repository, provide the repository URL if relevant, and provide specific details as to which raw blot/gel images, if any, are not available. Email us at plosone@plos.org if you have any questions.

3. Please include your tables as part of your main manuscript and remove the individual files. Please note that supplementary tables (should remain/ be uploaded) as separate ""supporting information"" files.

Additional Editor Comments :

The manuscript presents an investigation of MRSA isolated from commercial cheese. Reviewer #1 finds the manuscript generally good but recommends several major revisions, including formatting corrections, italic genes, and clarifications in the methodology and results sections. Reviewer #2 finds the manuscript covering important topic but raises significant methodological concerns, many missing data in many sections, the experimental design is simple, and test methods are routine.

The title does not comply with the subject of the study. Based on the reviewers' comments, the manuscript requires substantial revisions to address methodological concerns and improve clarity and formatting. Therefore, the editorial decision is to request a major revision. The authors should address the specific concerns raised by both reviewers.

[Note: HTML markup is below. Please do not edit.]

Reviewers' comments:

Reviewer's Responses to Questions

**Comments to the Author**

1. Is the manuscript technically sound, and do the data support the conclusions?

The manuscript must describe a technically sound piece of scientific research with data that supports the conclusions. Experiments must have been conducted rigorously, with appropriate controls, replication, and sample sizes. The conclusions must be drawn appropriately based on the data presented.

Reviewer #1: Yes

Reviewer #2: Partly

**********

2. Has the statistical analysis been performed appropriately and rigorously?

Reviewer #1: Yes

Reviewer #2: N/A

**********

3. Have the authors made all data underlying the findings in their manuscript fully available?

The PLOS Data policy requires authors to make all data underlying the findings described in their manuscript fully available without restriction, with rare exception (please refer to the Data Availability Statement in the manuscript PDF file). The data should be provided as part of the manuscript or its supporting information, or deposited to a public repository. For example, in addition to summary statistics, the data points behind means, medians and variance measures should be available. If there are restrictions on publicly sharing data—e.g. participant privacy or use of data from a third party—those must be specified.

Reviewer #1: Yes

Reviewer #2: No

**********

4. Is the manuscript presented in an intelligible fashion and written in standard English?

PLOS ONE does not copyedit accepted manuscripts, so the language in submitted articles must be clear, correct, and unambiguous. Any typographical or grammatical errors should be corrected at revision, so please note any specific errors here.

Reviewer #1: Yes

Reviewer #2: Yes

**********

5. Review Comments to the Author

Please use the space provided to explain your answers to the questions above. You may also include additional comments for the author, including concerns about dual publication, research ethics, or publication ethics. (Please upload your review as an attachment if it exceeds 20,000 characters)

Reviewer #1: Title: Molecular detection of super-antigenic Methicillin-Resistant Staphylococcus aureus from commercial cheese in Bangladesh

Comments to authors:

The authors of this study characterize the Staphylococcus aureus strains isolated from cheese samples, according to their antibiotic susceptibility patterns, virulence factors, biofilm formation ability and their genetic similarity. The study includes interesting data. However, some part of the manuscript must be summarized (introduction, discussion and conclusions). I have the following comments which the authors should address before the paper is considered for publication.

Line 51: correct, enterotoxin gene sea was present in 16.7% of isolates, while sec and TSST-1 were identified in 8.33%

Line 53: correct, were producers of slime or were slime producers

Line 115: correct, affinity for the most of β-lactams molecules

Line 115: correct, mecA, in italic

Line 137: complete the sentence “(SEa, SEb, SEc, SEd, SEe etc), and toxic shock syndrome toxin (TSST1)”.

Line 169: why you have incubated the collected samples at 37°C? I think that you must maintained these samples at 4°C before their microbiological analysis.

Line 170: correct, isolation and identification of S. aureus

Line 172: I think that 1g is not sufficient for the isolation of S. aureus, you must take from each sample 10 or 25g

Line 176: correct, mannitol salt agar is not specific only for S. aureus but this media is used to isolate the staphylococci genus.

Line 177: you must precise how number of suspected colonies were chosen from each positive sample?

Line 182: for the evaluation of S. aureus load in each sample, you must use another media, Baird Parker, and you must count the number of characteristic and non-characteristic S. aureus colonies. I think also that 1g is not sufficient to estimate the S. aureus load.

Line 192: correct, nuc, mecA in italic

Line 201: correct, In vitro in italic

Line 204: delete, biofilm

Line 210: correct, all the identified isolates

Line 210: correct, for staphylococcal enterotoxin genes (sea, seb, sec, sed, see)

Line 211: correct, the genes in italic (eta, etb, tst, icaA, icaB, icaC, icaD)

Line 218: correct, antimicrobial resistance testing

Line 221: correct, CLSI, 2020

Line 222: correct, disc diffusion method

Line 265-272: summarize this paragraph

Line 294: correct, sea, seb, sec, sed, and see

Line 305: correct, antibiotic susceptibility profile

Line 306-307: reformulate the sentence and you must indicate the multi-drug resistance phenotypes, which are observed in this study.

Line 327: correct, can causes severe

Line 337: correct, Haque et al. (2018),

Line 344: correct, in which S. aureus was isolated or recovered

Line 350: correct, low quality of raw materials

Line 380: correct, that enterotoxin genes sea and sec

Line 408: correct, due to a number of virulence factors

Line 415: correct, carry one or more staphylococcal enterotoxin genes

Line 420: reformulate, our research reported the frequency of TSST-1 with a value of 8.33%, which

Line 458: correct, the zoonotic trait of MRSA or zoonotic properties of MRSA

Line 500: correct, S. aureus may contaminate cheese or cheese may be contaminated with S. aureus, which is …

Reviewer #2: This paper reported the prevalence of Methicillin Susceptible S. aureus (MSSA) and Methicillin Resistance S. aureus (MRSA) in commercial cheese in Bangladesh. Meanwhile, antimicrobial resistance, virulence profiles, biofilm-forming capabilities were revealed. Additionally, the phylogenetic relationships of the obtained only one isolate with those from other countries were shown. This study covers a very important topic, especially since microbial resistance to previously known substances is increasing day by day, which puts lots of lives in danger of foodborne diseases.

However, the experimental design is simple, and test methods are routine.

Title: Upon reviewing the methods employed in the study, it is evident that demonstrating only the presence of genes is insufficient to justify using the term 'super-antigenic. It is also necessary to show that the toxins are produced and active. The validation of super-antigenic properties typically requires both genetic analyses and experimental methods assessing the biological activity of the toxins.

Therefore, merely indicating the presence of genes is not enough to definitively confirm the presence of super-antigenic properties. It would be appropriate to update the title in this context. Also, they should rearrange their result and discussion in this context.

The abstract does not adequately reflect the content of the study. The abstract outlines methods for the isolation of S. aureus, determination of antibiotic resistance profiles, and characterization of toxin genes. However, the findings also focus on biofilm formation, biofilm-associated genes, and antibiotic-resistance genes. In this context, the objectives and methods should be rewritten for greater clarity.

The introduction and discussion sections of the manuscript are too long and contain many repetitions. Please review and reorganize them. Especially, Introduction sectin can be shorten.

In material and methods and results section,Table 1-3 are missing.

ısolation step already been covering in 2.2. section. However the section 2.3 is more clear and better. The authors should merge these two sections, or omitted section 2.2.

The results section unnecessarily includes material and methods statements rather than the actual findings. The obtained results should be written. In other words, the results are very complex and not understandable, more information should be provided on study results, and material and methods sentences should be removed.

Specific comments:

Gene names should be italicized throughout the article.

Line 48-49: i) should be added "classical" before staphylococcal enterotoxins

ii) should be added "genes" after (TSST-1)

L49-50:according to which legislation? ıs it local EU or FDA?, and what are the CFU counts the safety thresholds, please clarify

L51: The authors mentioned Stapylococcal enterotoxins. However, the authors did not mention the detection of SE with ELISA or other techniques in both the abstract and material method sections. Please rephrase accordingly.

L 52-54: The sentence should be rearranged according to the percentages obtained from the methods used and the amount that is meant by the significant percentage should be stated., For example, Notably, 66.67% and 58.33% of the isolates exhibited biofilm formation based on the Congo Red and microtiter plate techniques, respectively, with significant percentages (?) carrying the icaA and icaD genes.

L59. key words: should be added genes after Staphylococcal Enterotoxins

L 170: Isolation and Identification of Organism >>>> Isolation ........ of S. aureus

L171: ISO 6579:2002(E) standard is for horizantal method for the Detection of Slamonella spp. Please check and rewrite the S. aureus standard.

L172: desired organisim>>>>should be changed as S. aureus

L173:which serial dilution? two or ten fold?

L171-192:ısolation step already been covering in 2.2. section. However the section 2.3 is more clear and better. The authors should merge these two sections, or omitted section 2.2.

L177-180:The sentences is not clear. Please check it and rewrite

L194: Staphylococcus aureus>>>> S. aureus

L196-197: Table 1 and Table 4 is missing.I could not see the tables in the article

L202 and L204: slim layer>>>> slime layer

L208:Title should be shortened as "Multiplex PCR for detection of virulence factor genes

L213: Table 2 and Table 3 İs missing

L215: for 45 minutes (Molecular Cloning: A Laboratory Manual. Joseph Sambrook, David W. Russell, 2001): please recheck this reference according to guidelines of journal

L221: CST>>>(CLSI 2020, Adjei et al., 2022)

L234: according to CLSI guidelines of 2020>>>>should be removed

L236:according to (Weinstein & Clinical and Laboratory Standards Institute, n.d.)>>>>should be removed

L241:how did you chose mecA positive isolates? How much isolates subject to squence analyses? ad how did you perform the sequence analysis?

L244-255: these sentences are not necessary. ıt should be removed

L265: Out of 36 samples tested? your sample size seems to 72 in abstract and material method section. You mentioned 36 samples tested in here. Please clarified.

L267-272:These sentences is not necessary, it should be removed.

L274:should be added the range of CFU count from analysed samples (min, max and average)

L282-290:This paragraph is too long and contains unnecessary information. This pharagraf should be rearragened like this, based on the PCR result, 100% of the total sample showed the presence of S. aureus, from which 33.33% of them were mecA positive

In other words, the number of S. aureus isolates and how many of them are MRSA should be clarified.

L291-304:did you found any enterotoxin genes and/or exfoliative genes? results are confusing please simplify and focus on prevelance of these genes.

L306-307: More information should be provided AMR profile of isolates. Also Table 3 is missing.

L301-312: These sentences are not necessary. ıt should be removed

L352:EC regulations (COMMISSION REGULATION (EC)?? Please check it and correct

L352: MIM et al., 2019>>>> Mim et al.

L378 and L501: Methicillin-resistant S. aureus>>> SHOULD BE WRİTE ONLY MRSA. Since you have previously mentioned the full name of the bacterium, it is sufficient to use only its abbreviation

L501:25%??? İs this rate for MRSA? This rate seems to be 33% for MRSA in results. Please check and correct.

fİGURE 8: The colors in the first image should be described to indicate what they represent.(What do the purple and its shades in the first image represent?)

**********

6. PLOS authors have the option to publish the peer review history of their article (what does this mean?). If published, this will include your full peer review and any attached files.

If you choose “no”, your identity will remain anonymous but your review may still be made public.

**Do you want your identity to be public for this peer review?** For information about this choice, including consent withdrawal, please see our Privacy Policy.

Reviewer #1: No

Reviewer #2: No

**********

[NOTE: If reviewer comments were submitted as an attachment file, they will be attached to this email and accessible via the submission site. Please log into your account, locate the manuscript record, and check for the action link "View Attachments". If this link does not appear, there are no attachment files.]

While revising your submission, please upload your figure files to the Preflight Analysis and Conversion Engine (PACE) digital diagnostic tool, https://pacev2.apexcovantage.com/. PACE helps ensure that figures meet PLOS requirements. To use PACE, you must first register as a user. Registration is free. Then, login and navigate to the UPLOAD tab, where you will find detailed instructions on how to use the tool. If you encounter any issues or have any questions when using PACE, please email PLOS at figures@plos.org. Please note that Supporting Information files do not need this step.

# Author comment: Author response to Decision Letter 1

**DOI:** 10.1371/journal.pone.0350222.r002

12 Jan 2025

Response to Reviewer

“Molecular detection of super-antigenic Methicillin-Resistant Staphylococcus aureus from commercial cheese in Bangladesh”

Dear Editor

I would like to express my gratitude to you for your kind handling of our manuscript. Due to some political issues in our country, and for that I could not reply within time.

First, I would like to thank the reviewers for their valuable comments and criticisms, which made our manuscript more scientific and pertinent. Please, below the point-by-point reply for their valuable comments.

Dear Reviewer 1:

First, we would like to disclose our thanks for your time to review our manuscript, and to provide scientific criticism on different aspects of our manuscript. May, I request you to have your kind attention again on the replies we provided below against your specific comments.

Reviewer: 1

The authors of this study characterize the Staphylococcus aureus strains isolated from cheese samples, according to their antibiotic susceptibility patterns, virulence factors, biofilm formation ability and their genetic similarity. The study includes interesting data. However, some part of the manuscript must be summarized (introduction, discussion and conclusions). I have the following comments which the authors should address before the paper is considered for publication.

Thank you very much for your appreciative comments on our submitted manuscript.

We have already condensed those sections, and we believe they are now sufficiently concise, enhancing the manuscript's quality.

Line 51 to Line 170: corrected.

Line 172: Thank you for your attention to detail. We apologize for the oversight, 10g samples were indeed used for S. aureus isolation, but this was mistakenly documented as 1g in the manuscript. In order to make our approach further clearer, we have fixed this mistake and included the reference that served as the basis for our sample strategy. We value your suggestions on how to make our work more accurate.

Line 176 and Line 182: We appreciate your observation. You are right; rather than being only specific for S. aureus, mannitol salt agar (MSA) is selective for the Staphylococcus genus. Because of its high salt content, which prevents non-staphylococci from growing, we selected MSA, and further biochemical testing verified that it was S. aureus. I have made changes to the manuscript, though, such that S. aureus is only mentioned upon PCR confirmation. We value your consideration of this particular detail, which has improved the precision of our technique explanation.

Line 177: I appreciate your insightful comments. From each positive sample, we chose a predetermined number of suspicious colonies based on their unique morphological traits on the selective media. To guarantee a uniform and representative study across samples, we have made the selection criteria for colonies clear in the updated manuscript. We value your recommendation to make our methods more transparent.

Line 192 to Line 500: Corrected

Dear reviewer, we are grateful for your valuable comments and critiques on our manuscript. We believe your comments are logical to address and will fit the journal. We have tried to address all the valuable comments.

………………………………………………………………………………………………………

Dear Reviewer 2:

First, we want to extend our gratitude to you for your valuable comments. May, I request you to have your kind attention again on the replies we provided below against your specific comments.

Reviewer: 2

This paper reported the prevalence of Methicillin Susceptible S. aureus (MSSA) and Methicillin Resistance S. aureus (MRSA) in commercial cheese in Bangladesh. Meanwhile, antimicrobial resistance, virulence profiles, and biofilm-forming capabilities were revealed. Additionally, the phylogenetic relationships of the obtained only one isolate with those from other countries were shown. This study covers a very important topic, especially since microbial resistance to previously known substances is increasing day by day, which puts lots of lives in danger of foodborne diseases. However, the experimental design is simple, and test methods are routine.

We appreciate your insightful remarks and your acknowledgment of the significance of our research on the prevalence of Methicillin-Resistant S. aureus in commercial cheese in Bangladesh. We all agree that antimicrobial resistance is a serious and escalating public health issue, and our goal was to provide insightful information on how it occurs in a food product that is often ingested. We take note of your observation that our experimental design is simple and that the test procedures are standard. Our goal was to conduct a foundational study to identify the presence of these strains, characterize their resistance profiles, and reveal initial insights into biofilm formation and phylogenetic relationships. Despite being routine, these techniques have been proven to be effective for preliminary prevalence research, and we think they offer crucial baseline information for comprehending the possible dangers of S. aureus in foodborne settings. This foundation can help expand on these findings through additional study employing more sophisticated or experimental methodologies.

Regarding our publication, "Molecular detection of super-antigenic Methicillin-Resistant Staphylococcus aureus from commercial cheese in Bangladesh," we appreciate your thoughtful criticism and helpful suggestions. We have carefully reviewed your recommendations and made the necessary changes to improve the manuscript's quality, rigor, and clarity.

Below, we provide detailed responses to each point raised:

1. Title Revision

Upon reviewing the methods employed in the study, it is evident that demonstrating only the presence of genes is insufficient to justify using the term 'super-antigenic. It is also necessary to show that the toxins are produced and active. The validation of super-antigenic properties typically requires both genetic analyses and experimental methods assessing the biological activity of the toxins.

Therefore, merely indicating the presence of genes is not enough to definitively confirm the presence of super-antigenic properties. It would be appropriate to update the title in this context. Also, they should rearrange their result and discussion in this context.

We acknowledge that demonstrating the mere presence of genes does not confirm super-antigenic properties. In order to appropriately convey the breadth of the work, we have revised the title to emphasize gene presence rather than active toxin generation or super-antigenicity.

2. Abstract Revision

The abstract does not adequately reflect the content of the study. The abstract outlines methods for the isolation of S. aureus, determination of antibiotic resistance profiles, and characterization of toxin genes. However, the findings also focus on biofilm formation, biofilm-associated genes, and antibiotic-resistance genes. In this context, the objectives and methods should be rewritten for greater clarity.

To make sure the abstract reflects the entire breadth of the study, we made revisions. We made the goals and procedures clearer, focusing on the characterization of antibiotic-resistant genes, biofilm formation, and biofilm-associated genes. This update provides a more thorough summary of the objectives and conclusions of the study.

3. Introduction and Discussion Revision

The introduction and discussion sections of the manuscript are too long and contain many repetitions. Please review and reorganize them. Especially, the Introduction section can be shortened.

We value your advice on this subject. To make the material more succinct and targeted, we have gone over both parts and eliminated repetitions, especially in the Introduction. We believe these revisions improve readability and maintain essential content.

4. Reorganization of Results and Discussion

Results and Discussion should be organized logically, particularly regarding super-antigenic gene findings.

To ensure a logical flow and enhance clarity, especially with regard to data pertaining to super-antigenic genes, we have restructured the Results and Discussion section. We believe the presentation is now more cohesive.

5. Materials and Methods Sections 2.2 and 2.3

Reviewer comment: In material and methods and results section, Table 1-3 are missing. ısolation step already been covering in 2.2. section. However the section 2.3 is more clear and better. The authors should merge these two sections, or omitted section 2.2. The results section unnecessarily includes material and methods statements rather than the actual findings. The obtained results should be written. In other words, the results are very complex and not understandable, more information should be provided on study results, and material and methods sentences should be removed.

We apologize for this omission. Now that Tables 1–3 are included in the text, they are all positioned correctly in the Materials, Methods, and Results sections. To eliminate duplication, we combined Sections 2.2 and 2.3, keeping the more lucid information that was formerly in Section 2.3. The Methods section has been simplified in this iteration, making it easier to understand. We have updated the Results section to only highlight study outcomes in response to your input. A straightforward presentation of the results was ensured by removing the Materials and Methods sections, and extra information was included to improve comprehension of our findings.

Line 48- Line 173: Corrected

Line 171- Line 192: We appreciate the suggestion. We provide the groundwork for comprehending the isolation in Section 2.2 by offering a thorough description of the organism's first identification procedure. On the other hand, Section 2.3 explains the methods for counting the microbial load and is especially concerned with the quantitative evaluation. We believe that by separating the identification and quantification processes, keeping these parts distinct preserves clarity.

Line 196- Line 197: Corrected

Line 202- Line 204: Corrected

Line 208: We have updated the title.

Line 213: Corrected.

Line 215-Line 236: Corrected.

Line 241: I appreciate you asking these questions. We made sure that only verified carriers were included by choosing mecA-positive isolates based on PCR validation. We performed sequence analysis on 10 of the 24 isolates in order to learn more about their genetic diversity. Sanger sequencing was used for the sequencing in order to guarantee precise and superior data. We prepared, sequenced, and analyzed these samples by conventional procedures, as explained in the Methods section.

Line 244- Line 272: Corrected.

Line 274: We appreciate the suggestion. The CFU count data is shown in two figures that clearly show the mean, standard deviation, and range. The purpose of these figures is to provide a thorough understanding of the distribution of data throughout the examined samples. Although we think this graphic depiction conveys the information well, we would be pleased to provide further explanation if necessary.

Line 282- Line 501: Updated and Corrected.

Dear reviewer, we are grateful for your valuable comments and critiques on our manuscript. We believe your comments are logical to address and will fit the journal. We have tried to address all the valuable comments.

Thanks again, and have a good day.

Best regards

Prof. Dr. Ferdaus Mohd Altaf HOSSAIN

# Peer review: Decision Letter 1

**DOI:** 10.1371/journal.pone.0350222.r003

**Authors:** Reham Mokhtar ELTarabili

10 Mar 2025

PONE-D-24-38694R1

Virulence Determinants and Toxin profile of Methicillin Resistant Staphylococcus aureus from Commercial Cheese in Bangladesh: A public Health Risk

PLOS ONE

Dear Dr. Hossain,

Thank you for submitting your manuscript to PLOS ONE. After careful consideration, we feel that it has merit but does not fully meet PLOS ONE’s publication criteria as it currently stands. Therefore, we invite you to submit a revised version of the manuscript that addresses the points raised during the review process.

==============================

Two reviewers assessed your manuscript. They provided a number of valid comments and suggestions that must be carefully addressed by the authors upon revision.

==============================

Please submit your revised manuscript by Apr 24 2025 11:59PM. If you will need more time than this to complete your revisions, please reply to this message or contact the journal office at plosone@plos.org. When you're ready to submit your revision, log on to https://www.editorialmanager.com/pone/ and select the 'Submissions Needing Revision' folder to locate your manuscript file.

Please include the following items when submitting your revised manuscript:

- A rebuttal letter that responds to each point raised by the academic editor and reviewer(s). You should upload this letter as a separate file labeled 'Response to Reviewers'.
- A marked-up copy of your manuscript that highlights changes made to the original version. You should upload this as a separate file labeled 'Revised Manuscript with Track Changes'.
- An unmarked version of your revised paper without tracked changes. You should upload this as a separate file labeled 'Manuscript'.

If you would like to make changes to your financial disclosure, please include your updated statement in your cover letter. Guidelines for resubmitting your figure files are available below the reviewer comments at the end of this letter.

If applicable, we recommend that you deposit your laboratory protocols in protocols.io to enhance the reproducibility of your results. Protocols.io assigns your protocol its own identifier (DOI) so that it can be cited independently in the future. For instructions see: https://journals.plos.org/plosone/s/submission-guidelines#loc-laboratory-protocols. Additionally, PLOS ONE offers an option for publishing peer-reviewed Lab Protocol articles, which describe protocols hosted on protocols.io. Read more information on sharing protocols at https://plos.org/protocols?utm_medium=editorial-email&utm_source=authorletters&utm_campaign=protocols.

We look forward to receiving your revised manuscript.

Kind regards,

Reham Mokhtar ELTarabili

Academic Editor

PLOS ONE

[Note: HTML markup is below. Please do not edit.]

Reviewers' comments:

Reviewer's Responses to Questions

**Comments to the Author**

1. If the authors have adequately addressed your comments raised in a previous round of review and you feel that this manuscript is now acceptable for publication, you may indicate that here to bypass the “Comments to the Author” section, enter your conflict of interest statement in the “Confidential to Editor” section, and submit your "Accept" recommendation.

Reviewer #1: All comments have been addressed

Reviewer #3: (No Response)

**********

2. Is the manuscript technically sound, and do the data support the conclusions?

The manuscript must describe a technically sound piece of scientific research with data that supports the conclusions. Experiments must have been conducted rigorously, with appropriate controls, replication, and sample sizes. The conclusions must be drawn appropriately based on the data presented.

Reviewer #1: Yes

Reviewer #3: No

**********

3. Has the statistical analysis been performed appropriately and rigorously?

Reviewer #1: Yes

Reviewer #3: N/A

**********

4. Have the authors made all data underlying the findings in their manuscript fully available?

The PLOS Data policy requires authors to make all data underlying the findings described in their manuscript fully available without restriction, with rare exception (please refer to the Data Availability Statement in the manuscript PDF file). The data should be provided as part of the manuscript or its supporting information, or deposited to a public repository. For example, in addition to summary statistics, the data points behind means, medians and variance measures should be available. If there are restrictions on publicly sharing data—e.g. participant privacy or use of data from a third party—those must be specified.

Reviewer #1: Yes

Reviewer #3: (No Response)

**********

5. Is the manuscript presented in an intelligible fashion and written in standard English?

PLOS ONE does not copyedit accepted manuscripts, so the language in submitted articles must be clear, correct, and unambiguous. Any typographical or grammatical errors should be corrected at revision, so please note any specific errors here.

Reviewer #1: Yes

Reviewer #3: (No Response)

**********

6. Review Comments to the Author

Please use the space provided to explain your answers to the questions above. You may also include additional comments for the author, including concerns about dual publication, research ethics, or publication ethics. (Please upload your review as an attachment if it exceeds 20,000 characters)

Reviewer #1: Title: Molecular detection of super-antigenic Methicillin-Resistant Staphylococcus aureus from commercial cheese in Bangladesh

Comments to authors:

The authors of this paper characterize the Staphylococcus aureus isolates from cheese samples in Bangladesh, according to their antibiotic resistance patterns, enterotoxin gene profiles and their biofilm formation ability. The result of this study is interesting and should be of interest to the readership of your journal. I have the following observations which the authors should address before the paper is considered for publication.

Line 45: correct, comprehensive analysis

Line 48: correct, staphylococcal enterotoxin genes (sea, seb, sec, sed and see)

Line 51: correct, enterotoxin gene sea……………., while sec……………..

Line 59: correct, staphylococcal enterotoxin genes

Line 95: correct, during handling

Line 115: correct, for β-lactams

Line 115: correct, mecA in italic

Line 137-138: complete the sentence “(SEa, SEb, SEc, SEd, SEe etc), and toxic shock syndrome toxin (TSST”

Line 151: correct, by …..

Line 172: why you have sampled only 1g from each sample? . You must sample at least 10g

Line 176: correct, the mannitol salt agar is not specific for S. aureus species but specific for the Staphylococcus genus.

Line 177: you must precise the number of colonies chosen from each positive sample

Line 186: correct, Pétri plate

Line 201: correct, in vitro in italic

Line 210: correct, for staphylococcal enterotoxin genes (sea, seb,…….)

Line 210-211: you must put the targeted genes in italic

Line 223: why you have used the PBS for the preparation of the bacterial suspension?

Line 235: correct, and the strains were classified as susceptible, …..

Line 264: with the biochemical tests, you can not confirmed the S. aureus species, as you have many Staphylococcus species which are coagulase positive. For this, you must perform other tests and confirm the obtained isolates with molecular method (PCR).

Line 293: correct, All of the MRSA-positive isolates

Line 294: correct, such as sea, seb, sec, …..

Line 330: correct not for all but for the most β-lactam molecules

Line 340: correct, the results of this study do not agree with agree with those of Ifra …..

Line 413: correct, genes sea and sec

Line 421: correct, in S. aureus isolates from Minas ……

Line 424: you can compare your results with those obtained by TITOUCHE et al (2024). Investigation of Biofilm Formation Ability and Antibiotic Resistance of Staphylococcus aureus Isolates from Food Products.

Line 455: correct, however, the presence of these genes in S. aureus isolates from cheese samples

Line 500: correct, cheese may be contaminated with S. aureus…..

Line 504: correct, to decrease the contamination rate of

Reviewer #3: The authors performed this study to determine the virulence and toxin profiles of methicillin-resistant Staphylococcus aureus isolated from commercial cheese in Bangladesh. This type of study is really important for public health. Unfortunately, this study has many serious drawbacks. I found many discrepancies in the study design, especially in antibiotic selection. I believe the authors should check the CLSI guidelines properly before selecting any antibiotics. Also, I believe the authors need to perform the study again. However, please find my comments below.

Line 120: How much distilled water? Did you make the distilled water sterile before using it?

Line 145: Reference for boiling method?

Tables 2 and 5: I recommend using these tables as supplementary material.

Line 162: “2.6 Multiplex PCR for detection of virulence factor genes” – but you also mentioned resistance genes here. Please modify the name of the section.

Also, did you check resistance genes before determining their phenotypic resistance profiles? Please clarify this.

Line 176-195:

- So, you used oxacillin for S. aureus, right? How did you interpret the data based on CLSI 2020? You have to check their MIC; you can’t come to an outcome for oxacillin using the disk diffusion test. CLSI 2020 clearly mentions that oxacillin disk testing is not reliable for S. aureus. I believe this is a serious drawback of your study. You should go through your study again.

- Moreover, how did you interpret the resistance profiles of ampicillin and amoxicillin? As far as I know, you can only use penicillin (10 units) for S. aureus, which is another drawback.

- The same is true for streptomycin, cefotaxime, cefuroxime, and nalidixic acid. You should check the CLSI 2020 guidelines before using any antibiotics.

Line 189: “Gentamycin” should be “Gentamicin.” Please correct it where needed.

Line 200: “mecA” should be in italics. All the genes’ names should be in italics. Please correct it where needed.

Line 198-207: References for any software or methods you used are missing.

Line 209-214:

- I was just wondering why you used different versions of GraphPad Prism in the same study.

- “P” from “P-value” should be in italics.

- “study's findings and the authority” – what does that mean?

- You don’t need to provide the prevalence equation.

Line 246: You can’t determine MRSA by using only the mecA gene. There are several resistance genes that can also be responsible for MRSA, e.g., mecB, mecC, mecD, or others. This is another drawback of this study.

**********

7. PLOS authors have the option to publish the peer review history of their article (what does this mean?). If published, this will include your full peer review and any attached files.

If you choose “no”, your identity will remain anonymous but your review may still be made public.

**Do you want your identity to be public for this peer review?** For information about this choice, including consent withdrawal, please see our Privacy Policy.

Reviewer #1: No

Reviewer #3: **Yes:** Md Saiful Islam

**********

[NOTE: If reviewer comments were submitted as an attachment file, they will be attached to this email and accessible via the submission site. Please log into your account, locate the manuscript record, and check for the action link "View Attachments". If this link does not appear, there are no attachment files.]

While revising your submission, please upload your figure files to the Preflight Analysis and Conversion Engine (PACE) digital diagnostic tool, https://pacev2.apexcovantage.com/. PACE helps ensure that figures meet PLOS requirements. To use PACE, you must first register as a user. Registration is free. Then, login and navigate to the UPLOAD tab, where you will find detailed instructions on how to use the tool. If you encounter any issues or have any questions when using PACE, please email PLOS at figures@plos.org. Please note that Supporting Information files do not need this step.

# Author comment: Author response to Decision Letter 2

**DOI:** 10.1371/journal.pone.0350222.r004

27 Apr 2025

Response to Reviewer

“Virulence Determinants and Toxin Profile of Methicillin-Resistant Staphylococcus aureus from Commercial Cheese in Bangladesh: A Public Health Risk”

Dear Editor

I would like to express my gratitude to you for your kind handling of our manuscript. First, I would like to thank the reviewers for their valuable comments and criticisms, which made our manuscript more scientific and pertinent. It took us a bit long time to submit our feedback because, according to our reviewer's comment, we performed some parts of the experiment again and tried to trace back so that we could resolve the shortcomings of our study.

I'm asking the reviewers to look at the detailed response for their insightful feedback, which is listed below.

Dear Reviewer 1:

First, we would like to express our gratitude for your time to review our manuscript and for providing scientific criticism on different aspects of our manuscript. May I request you to have your kind attention again on the replies we provided below against your specific comments.

Reviewer: 1

The authors of this paper characterize the Staphylococcus aureus isolates from cheese samples in Bangladesh, according to their antibiotic resistance patterns, enterotoxin gene profiles and their biofilm formation ability. The result of this study is interesting and should be of interest to the readership of your journal.

Thank you very much for your appreciative comments on our submitted manuscript.

Line 45 to Line 151: corrected.

Line 172: Why you have sampled only 1g from each sample? You must sample at least 10g.

Thank you for your attention to detail. Already corrected and the reply was submitted to the first reviewing process.

Line 176 and Line 177: Thank you for your attention to detail. Already corrected and the reply was submitted to the first reviewing process.

Line 177: you must precise the number of colonies chosen from each positive sample.

Thank you for your valuable suggestion. We have now clearly mentioned the number of colonies (2-3 colonies) from each culture-positive sample.

Line 186- Line 211: Already corrected in the first reviewing process.

Line 223: why you have used the PBS for the preparation of the bacterial suspension?

Thank you for your insightful comment. Phosphate-buffered saline (PBS) is used in cell culture for several key reasons, primarily to maintain osmotic balance and pH, ensuring cell viability and integrity during various procedures. It's an isotonic solution, meaning its salt concentration is similar to that of cells, preventing them from shrinking or bursting due to water movement.

Line 235: Corrected

Line 264: With the biochemical tests, you can not confirm the S. aureus species, as you have many Staphylococcus species which are coagulase positive. For this, you must perform other tests and confirm the obtained isolates with molecular method (PCR).

Thank you for your valuable observation. We concur that since there are other coagulase-positive Staphylococcus species, biochemical testing by alone is insufficient to confirm Staphylococcus aureus. Since the main focus of our investigation was molecular detection, PCR targeting the particular nuc gene was employed to confirm the presence of S. aureus.

Line 293 to Line 421: Corrected.

Line 424: you can compare your results with those obtained by TITOUCHE et al (2024). Investigation of Biofilm Formation Ability and Antibiotic Resistance of Staphylococcus aureus Isolates from Food Products.

Thank you for your suggestion. We added the comparison in our discussion part.

Line 455 to Line 504: Corrected.

Dear reviewer, we are grateful for your valuable comments and critiques on our manuscript. We believe your comments are logical to address and will fit the journal. We have tried to address all the valuable comments.

………………………………………………………………………………………………………

Dear Reviewer 3:

First, we want to extend our gratitude to you for your valuable comments. May I request you to have your kind attention again on the replies we provided below against your specific comments.

Reviewer: 3

The authors performed this study to determine the virulence and toxin profiles of methicillin-resistant Staphylococcus aureus isolated from commercial cheese in Bangladesh. This type of study is really important for public health. Unfortunately, this study has many serious drawbacks. I found many discrepancies in the study design, especially in antibiotic selection. I believe the authors should check the CLSI guidelines properly before selecting any antibiotics. Also, I believe the authors need to perform the study again.

We appreciate your insightful remarks and your acknowledgment of the significance of our research on the prevalence of Methicillin-Resistant S. aureus in commercial cheese in Bangladesh. We acknowledge the concerns regarding antibiotic selection and have taken your suggestions seriously. We have already reassessed our approach and conducted this part of the study following CLSI recommendations to address this issue. We think this has increased the accuracy of our investigation and reinforced our findings. We sincerely value your helpful criticism, which has enabled us to improve our work and guarantee its applicability to public health.

Regarding our publication, “Virulence Determinants and Toxin Profile of Methicillin-Resistant Staphylococcus aureus from Commercial Cheese in Bangladesh: A Public Health Risk”, we appreciate your thoughtful criticism and helpful suggestions. We have carefully reviewed your recommendations and made the necessary changes to improve the manuscript's quality, rigor, and clarity.

Below, we provide detailed responses to each point raised:

Line 120: How much distilled water? Did you make the distilled water sterile before using it?

Thank you for your critical evaluation. We have utilized 90 ml of distilled water, which was sterilized by autoclaving it properly at 121 °C and 15 PSI pressure. We have revised our writing regarding this.

Line 145: Reference for boiling method?

We have updated the reference section.

Tables 2 and 5: I recommend using these tables as supplementary material.

I appreciate your recommendation on Tables 2 and 5. We concur that both tables, which provide information on thermal cycling conditions, could be better suited as supplemental content to help the main text flow. Following this, we have updated the manuscript and added them as supplemental tables.

Line 162: “2.6 Multiplex PCR for detection of virulence factor genes” – but you also mentioned resistance genes here. Please modify the name of the section.

Also, did you check resistance genes before determining their phenotypic resistance profiles? Please clarify this.

Thank you for pointing this out. We have updated the section heading. Regarding your second point, we performed a phenotypic resistance profile before detecting resistance genes via PCR. We apologize for the confusion and have clarified this sequence in the revised manuscript.

Line 176-195:

- So, you used oxacillin for S. aureus, right? How did you interpret the data based on CLSI 2020? You have to check their MIC; you can’t come to an outcome for oxacillin using the disk diffusion test. CLSI 2020 clearly mentions that oxacillin disk testing is not reliable for S. aureus. I believe this is a serious drawback of your study. You should go through your study again.

- Moreover, how did you interpret the resistance profiles of ampicillin and amoxicillin? As far as I know, you can only use penicillin (10 units) for S. aureus, which is another drawback.

- The same is true for streptomycin, cefotaxime, cefuroxime, and nalidixic acid. You should check the CLSI 2020 guidelines before using any antibiotics.

I appreciate your insightful comments. Your thorough analysis and insightful remarks about the antibiotic susceptibility testing are greatly appreciated.

In compliance with the CLSI 2020 recommendations, I have carefully re-evaluated the antibiotic selection and interpretation criteria after going over the pertinent portion of my study again. In light of this, I updated the antibiotic panel to include 13 agents that are suitable and advised for Staphylococcus aureus in accordance with CLSI guidelines. In particular:

-Following CLSI 2020, I have excluded oxacillin disc diffusion findings, recognising that MIC testing is the proper technique for interpreting oxacillin in S. aureus.

-Additionally, I have reinterpreted ampicillin and amoxicillin and now only use penicillin (10 units) for S. aureus β-lactam susceptibility profiling.

-Similarly, I have removed the antibiotics that CLSI does not advise for S. aureus susceptibility testing, including streptomycin, cefotaxime, cefuroxime, and nalidixic acid.

These corrections have been incorporated into the revised dataset and analysis, ensuring full alignment with the CLSI 2020 standards. I believe these updates address the concerns raised and improve the overall reliability of the study.

Line 189- Line 214: Updated and Corrected.

Line 246: You can’t determine MRSA by using only the mecA gene. There are several resistance genes that can also be responsible for MRSA, e.g., mecB, mecC, mecD, or others. This is another drawback of this study.

Regarding MRSA detection, I appreciate your insightful assessment. As you correctly point out, although the mecA gene is most frequently linked to methicillin resistance in Staphylococcus aureus, other mec homologs, including mecB, mecC, and mecD, have also been found to be involved in the MRSA phenotype.

Since mecA is still the most common and extensively researched marker for MRSA identification, especially in clinical and epidemiological settings, we concentrated on it in this investigation. I do admit, though, that mecA alone might not be able to identify all possible MRSA strains, particularly uncommon or newly discovered varieties. In order to present a more impartial analysis of the findings, I will address this significant constraint in the discussion section.

Dear reviewer, we are grateful for your valuable comments and critiques on our manuscript. We believe your comments are logical to address and will fit the journal. We have tried to address all the valuable comments.

Thanks again, and have a good day.

Best regards

Prof. Dr. Ferdaus Mohd Altaf HOSSAIN

Attachment Submitted filename: Response to Reviewer on 2nd review.docx

# Peer review: Decision Letter 2

**DOI:** 10.1371/journal.pone.0350222.r005

**Authors:** Abayeneh Girma

18 Aug 2025

PONE-D-24-38694R2

Virulence Determinants and Toxin profile of Methicillin Resistant Staphylococcus aureus from Commercial Cheese in Bangladesh: A public Health Risk

PLOS ONE

Dear Dr. Hossain,

Thank you for submitting your manuscript to PLOS ONE. After careful consideration, we feel that it has merit but does not fully meet PLOS ONE’s publication criteria as it currently stands. Therefore, we invite you to submit a revised version of the manuscript that addresses the points raised during the review process.

Please submit your revised manuscript by Oct 02 2025 11:59PM. If you will need more time than this to complete your revisions, please reply to this message or contact the journal office at plosone@plos.org. When you're ready to submit your revision, log on to https://www.editorialmanager.com/pone/ and select the 'Submissions Needing Revision' folder to locate your manuscript file.

Please include the following items when submitting your revised manuscript:

- A rebuttal letter that responds to each point raised by the academic editor and reviewer(s). You should upload this letter as a separate file labeled 'Response to Reviewers'.
- A marked-up copy of your manuscript that highlights changes made to the original version. You should upload this as a separate file labeled 'Revised Manuscript with Track Changes'.
- An unmarked version of your revised paper without tracked changes. You should upload this as a separate file labeled 'Manuscript'.

If you would like to make changes to your financial disclosure, please include your updated statement in your cover letter. Guidelines for resubmitting your figure files are available below the reviewer comments at the end of this letter.

If applicable, we recommend that you deposit your laboratory protocols in protocols.io to enhance the reproducibility of your results. Protocols.io assigns your protocol its own identifier (DOI) so that it can be cited independently in the future. For instructions see: https://journals.plos.org/plosone/s/submission-guidelines#loc-laboratory-protocols. Additionally, PLOS ONE offers an option for publishing peer-reviewed Lab Protocol articles, which describe protocols hosted on protocols.io. Read more information on sharing protocols at https://plos.org/protocols?utm_medium=editorial-email&utm_source=authorletters&utm_campaign=protocols.

We look forward to receiving your revised manuscript.

Kind regards,

Abayeneh Girma

Academic Editor

PLOS ONE

Journal Requirements:

If the reviewer comments include a recommendation to cite specific previously published works, please review and evaluate these publications to determine whether they are relevant and should be cited. There is no requirement to cite these works unless the editor has indicated otherwise.

[Note: HTML markup is below. Please do not edit.]

Reviewers' comments:

Reviewer's Responses to Questions

**Comments to the Author**

1. If the authors have adequately addressed your comments raised in a previous round of review and you feel that this manuscript is now acceptable for publication, you may indicate that here to bypass the “Comments to the Author” section, enter your conflict of interest statement in the “Confidential to Editor” section, and submit your "Accept" recommendation.

Reviewer #4: All comments have been addressed

Reviewer #5: (No Response)

Reviewer #6: All comments have been addressed

Reviewer #7: (No Response)

Reviewer #8: All comments have been addressed

**********

2. Is the manuscript technically sound, and do the data support the conclusions?

The manuscript must describe a technically sound piece of scientific research with data that supports the conclusions. Experiments must have been conducted rigorously, with appropriate controls, replication, and sample sizes. The conclusions must be drawn appropriately based on the data presented.

Reviewer #4: Partly

Reviewer #5: No

Reviewer #6: Yes

Reviewer #7: Partly

Reviewer #8: Yes

**********

3. Has the statistical analysis been performed appropriately and rigorously?

Reviewer #4: No

Reviewer #5: N/A

Reviewer #6: Yes

Reviewer #7: N/A

Reviewer #8: Yes

**********

4. Have the authors made all data underlying the findings in their manuscript fully available?

The PLOS Data policy requires authors to make all data underlying the findings described in their manuscript fully available without restriction, with rare exception (please refer to the Data Availability Statement in the manuscript PDF file). The data should be provided as part of the manuscript or its supporting information, or deposited to a public repository. For example, in addition to summary statistics, the data points behind means, medians and variance measures should be available. If there are restrictions on publicly sharing data—e.g. participant privacy or use of data from a third party—those must be specified.

Reviewer #4: Yes

Reviewer #5: Yes

Reviewer #6: Yes

Reviewer #7: Yes

Reviewer #8: Yes

**********

5. Is the manuscript presented in an intelligible fashion and written in standard English?

PLOS ONE does not copyedit accepted manuscripts, so the language in submitted articles must be clear, correct, and unambiguous. Any typographical or grammatical errors should be corrected at revision, so please note any specific errors here.

Reviewer #4: No

Reviewer #5: No

Reviewer #6: Yes

Reviewer #7: Yes

Reviewer #8: Yes

**********

6. Review Comments to the Author

Please use the space provided to explain your answers to the questions above. You may also include additional comments for the author, including concerns about dual publication, research ethics, or publication ethics. (Please upload your review as an attachment if it exceeds 20,000 characters)

Reviewer #4: Virulence Determinants and Toxin profile of Methicillin Resistant Staphylococcus aureus

from Commercial Cheese in Bangladesh: A public Health Risk

PONE-D-24-38694_R2_reviewer

Comments on Revised version:

Abstract:

Line 28-30: In my opinion, the authors could represent this portion in another way. Suddenly started about MRSA in the very next sentence. The relationship of MRSA and cheese might come first and then objective of the research could be mentioned.

Line 35: Please rephrase this sentence.

Line 36: please write as……….. polymerase chain reaction (PCR)

Line 41-42: Please mention the safety threshold briefly.

Introduction:

Authors did not establish link between cheese and MRSA.

No research gap was mentioned.

Previous research on MRSA and cheese on global or national contexts was not mentioned.

Sources of contamination with MRSA should be discussed clearly.

Line 57-58: Please give reference.

Line 60-61: need reference(s).

Line 67-68: need reference(s)

Line 69-70: need reference(s).

Line 57-68: Need multiple references for each statement.

Materials and Methods:

Line `112-113: Only one raw milk cheese included? Why? Making comparison was not difficult?

Line 140-141: mention the rationale of using pour plate method.

Line 148-149: Why so many references for boiling method? Single reference was not enough? Why boiling method? Why did not any Kit?

Line 175: why CLSI, 2020? Why not recent?

Line 194-203: Use references.

Results:

As these findings were reviewed by previous reviewers, I don’t have queries.

Discussion:

Why 100%? Most of the authors found lower percentage of Staph. Aureus in their researches. The authors mentioned country and production procedure variations. But the commercial cheese production of renowned Brands follows the International standards, even in Bangladesh. In my opinion, authors should establish scientific basis of this finding.

As all samples exceeded the EU recommended standard, then what could be the authors decision about the commercial cheese in Bangladesh?

Conclusion:

Conclusion became too long. Should be more precise and shorter.

Line 427: After finishing the whole experiments, showing all findings, why did authors mention “may be’?

Reviewer #5: The manuscript entitled “Virulence Determinants and Toxin profile of Methicillin Resistant Staphylococcus aureus from Commercial Cheese in Bangladesh: A public Health Risk” presented the virulence and toxin factors of MRSA from commercial cheese in Bangladesh. The Authors also showed the AMR profile of MRSA by both phenotypic and genotypic approach. The manuscript has great importance in the field of AMR and food safety. However, the manuscript requires some corrections. There are areas of improvement. The knowledge gap should include why the study is different from other studies in Bangladesh. The discussion section has some repetitions. Please check the whole manuscript for English corrections. There is a serious issue in the methodology of bacterial load determination. All are given bellow-

Title: Why the word “public” starts with the small “p”.

Line no. 42: Please add “and” before “Food Safety and Standards Authority India (FSSAI)”.

Line no. 46: showed a moderate level of resistance. Check the sentence.

The gene name should be Italic. Check the uniformity of gene and species names throughout the manuscript.

Please check the whole manuscript for similar minor errors to improve the quality of the manuscript.

Line no. 110: Are all the brands from Bangladesh or imported as well?

Line 134-137: As per standard, the sample amount should be 10 grams. Thereafter, for ten-fold dilution, you must have to maintain 1:10 that is one part sample and 9 parts diluent. However, the Authors have not maintained this ratio. They used one gram sample and 900 microliter (0.9 ml) diluent that is 1: 0.9 ratio. your total volume is approximately 1.9mL. Your dilution factor is 1/1.9, which is approximately 10⁻⁰·²⁸, not 10⁻¹.

It is a serious mistake in the sample preparation and it will misinterpret the findings.

Line no. 150-151: Table 01, contains primers of nuc, mecA, and toxin genes.

Line no. 156: Table 01. Where is the supplementary table? After Table 1, where is Table 02?

Line no. 162-163: The sentence is not clear. Please mention the name of the test with the interpretation criteria.

Line no. 169-170: Approximate concentration of 1.5*10-8 CFU ml-1.

Line no. 247: total samples.

Line no. 380-382: Please mention the causes of differences in occurrences of MRSA in cheese particularly for Bangladesh.

Reviewer #6: Line 95: write the abbreviated form of International Institute for Diarrheal Disease Research within bracket

Line 132: change the heading 2.3 (Microbiological evaluation of Cheese sample). Aren’t the isolation, identification, molecular detection and antibiotic sensitivity test included in microbiological evaluation? Your writing resembles that only CFU counting represents the microbiological evaluation. You can change the heading as ‘’ total count of Staphylococcus genus’’.

Line 163: What are the modifications?

Line 213: Recheck this heading and make it shorter

Line 228: This study performed in Bangladesh. That’s why it is ok to compare the findings with the recommended limit of BFSA. But why the findings are compared to that of Standards Authority of India, and the European Union bypassing other countries like USA, Australia and others?

Line 228-229: Reference?

Line 286-287: 3.5 Toxin, Virulence, and antibiotic-resistant genes profile of Staphylococcus aureus isolated from cheese: What about SEb and SEd. Clarify their percentage.

Figure 6: it will be easier to understand if you represent the two pictures of PCR with two different figure names

Figure 8: same as figure 6

Figure 9: Try to increase the sharpness and clarity of the given diagram.

Line 341: Why is a matter of concern?

Discussion: line 388-394: Recheck the sequence and organization of the provided information. At first you wrote about the percentage of S. aureus and compared it with the existing data, then wrote about MRSA in the same manner. After that you repeated the S. aureus again. Try to write in more organized way.

Line 437: is it TEM or blaTEM?

Overall evaluation: Recheck the whole manuscript again for grammatical errors. The organism’s name and genes name must be in italic form. The headings under different section should be rechecked to make it simpler, smarter and shorter.

Reviewer #7: I have reviewed the manuscript "Virulence Determinants and Toxin Profile of Methicillin-Resistant Staphylococcus aureus from Commercial Cheese in Bangladesh: A Public Health Risk" for the first time. The authors have justified or corrected the observations made by reviewers in previous rounds. However, I believe there are still several errors or clarifications that need to be made, particularly in the methods and results.

Material and Methods

• Sections 2.4, 2.5, 2.6 and 2.7: Authors must include and mention the use of positive and negative controls.

• Table 1: The primers listed are not compatible with those used in the references indicated. Particularly in refs. 16 and 17, the primers are completely different. In the other cases (ref. 18), there are differences in some nucleotides. Authors should verify each primer in Tables 1, 3, and 4.

• Section 2.4/Line 148: It is excessive to use 5 references to cite the boiling method. Reduce to 1 or a maximum of 2.

• Section 2.6/Line 167: Authors must appropriately cite the CLSI manual used and not any other indirect reference.

• Sections 2.4 + 2.7: I recommend merging these two sections, as they are all PCR-based. Also, merge tables (1, 3, and 4)—note that Table 2 does not exist—and maintain the separation of primer groups by category as they were.

• These analyses are inappropriate and should be withdrawn. The fragment considered (233 bp) is very small and highly conserved in S. aureus (I blasted the OR096215 sequence and found nearly 2,000 sequences with 100% identity). The resolution of the phylogenetic tree does not allow for appropriate inferences between identical sequences. Any discussion or conclusion based on this analysis must be withdrawn.

Results:

• Since all PCR assays are from references and were not developed in the present study, I consider the gel figures (Figs. 3, 4, 6, 7, 8, 10B) unnecessary in the main body of the manuscript. I suggest transferring them as supplementary material.

• Figure 3: The authors indicate an expected amplification size of 280 bp for the nuc gene, but the figure shows bands above 300 bp. What is the reason for this?

• Figure 4: In the legend, the authors indicate that the samples in lanes 5 and 6 are negative. However, the same positive band can be seen in lanes 1 through 4.

• 3.4: The authors refer to Table 4, which currently lists primers against biofilm-producing genes. Verify.

• Figure 8: In panels A and B, the authors indicate other sizes such as "100 bp." Since the 100 band is not visible, I suggest only identifying the 500 band, which is the most intense. In panel B, something similar occurs as in Figure 3. The authors indicate that the detection band is 397 bp, however, in the gel photo, it appears to be below 300 bp. Is it possible that electrophoresis generated a curve? In any case, the use of positive controls is necessary to validate these results.

• Finally, I suggest a review of the language to avoid overly colloquial idioms and phrases.

Reviewer #8: The article is interesting and congratulations for your work. The authors did almost all required revisions.

**********

7. PLOS authors have the option to publish the peer review history of their article (what does this mean?). If published, this will include your full peer review and any attached files.

If you choose “no”, your identity will remain anonymous but your review may still be made public.

**Do you want your identity to be public for this peer review?** For information about this choice, including consent withdrawal, please see our Privacy Policy.

Reviewer #4: No

Reviewer #5: **Yes:** Md. Abdus Sobur

Reviewer #6: No

Reviewer #7: No

Reviewer #8: **Yes:** Naglaa S. Elabd

**********

[NOTE: If reviewer comments were submitted as an attachment file, they will be attached to this email and accessible via the submission site. Please log into your account, locate the manuscript record, and check for the action link "View Attachments". If this link does not appear, there are no attachment files.]

While revising your submission, please upload your figure files to the Preflight Analysis and Conversion Engine (PACE) digital diagnostic tool, https://pacev2.apexcovantage.com/. PACE helps ensure that figures meet PLOS requirements. To use PACE, you must first register as a user. Registration is free. Then, login and navigate to the UPLOAD tab, where you will find detailed instructions on how to use the tool. If you encounter any issues or have any questions when using PACE, please email PLOS at figures@plos.org. Please note that Supporting Information files do not need this step.

Attachment Submitted filename: plos one.docx

# Author comment: Author response to Decision Letter 3

**DOI:** 10.1371/journal.pone.0350222.r006

24 Feb 2026

Response to Reviewer

PONE-D-24-38694R2

“Virulence Determinants and Toxin Profile of Methicillin-Resistant Staphylococcus aureus from Commercial Cheese in Bangladesh: A Public Health Risk”

Dear Editor

I sincerely appreciate your kind handling of our manuscript. I am also grateful to the reviewers for their insightful comments and constructive criticisms, which have significantly improved the scientific rigor and relevance of our work. The revision process required additional time, as we repeated certain experiments and carefully re-examined aspects of the study in order to address the reviewers’ concerns and resolve identified limitations.

I'm asking the reviewers to look at the detailed response for their insightful feedback, which is listed below.

Dear Reviewer 4:

First, we would like to express our gratitude for your time to review our manuscript and for providing scientific criticism on different aspects of our manuscript. May I request you to have your kind attention again on the replies we provided below against your specific comments.

Reviewer: 4

Abstract:

Line 28-30: In my opinion, the authors could represent this portion in another way. Suddenly started about MRSA in the very next sentence. The relationship of MRSA and cheese might come first and then objective of the research could be mentioned.

Thank you for your constructive suggestion. We have revised Lines 28–30 to first establish the relationship between MRSA and cheese, followed by a clear statement of the study objective. The flow of the text has been improved accordingly.

Line 35: Please rephrase this sentence.

Thank you for your comment. The sentence in Line 35 has been rephrased for improved clarity and readability.

Line 36: please write as……….. polymerase chain reaction (PCR)

Thank you for your suggestion. The abbreviation has now been correctly written as polymerase chain reaction (PCR) in Line 36.

Line 41-42: Please mention the safety threshold briefly.

Thank you for your valuable comment. The relevant safety threshold has now been briefly mentioned in Lines 41–42.

Introduction:

Authors did not establish link between cheese and MRSA.

No research gap was mentioned.

Previous research on MRSA and cheese on global or national contexts was not mentioned.

Sources of contamination with MRSA should be discussed clearly.

Thank you for these insightful comments. The Introduction section has been substantially revised to:

• Clearly establish the link between cheese and MRSA,

• Highlight the existing research gap,

• Incorporate relevant global and national studies on MRSA in cheese, and

• Clearly discuss possible sources of MRSA contamination.

Line 57-58: Please give reference.

Appropriate references have now been added to support the statement in Lines 57–58.

Line 60-61: need reference(s).

Relevant references have been added to Lines 60–61.

Line 67-68: need reference(s)

Supporting references have now been included for the statement in Lines 67–68.

Line 69-70: need reference(s).

Necessary references have been added to Lines 69–70.

Line 57-68: Need multiple references for each statement.

Thank you for your valuable observation. Multiple appropriate references have now been added for each statement between Lines 57–68 to strengthen the scientific support.

Materials and Methods:

Line `112-113: Only one raw milk cheese included? Why? Making comparison was not difficult?

Thank you for your thoughtful comment. In the study area, only one type of cheese produced from raw milk was available during the sampling period. Therefore, inclusion of additional raw milk cheese types and comparative analysis was not feasible.

Line 140-141: mention the rationale of using pour plate method.

Thank you for your comment. We apologize for the confusion. The streaking method was used instead of the pour plate method.

Line 148-149: Why so many references for boiling method? Single reference was not enough? Why boiling method? Why did not any Kit?

Thank you for raising this important point. The number of references has been reduced to the most relevant one. The boiling method was chosen due to its cost-effectiveness, simplicity, and suitability for resource-limited laboratory settings without compromising PCR performance. However, we performed the extraction of genomic DNA using kit again.

Line 175: why CLSI, 2020? Why not recent?

Thank you for your comment. We apologize for the oversight. Antimicrobial susceptibility testing (AST) was performed following the most recent CLSI 2025 guidelines, and this has now been corrected and clearly stated in Line 175.

Line 194-203: Use references.

Appropriate and relevant references have now been added to support the methodology described in Lines 194–203.

Results:

As these findings were reviewed by previous reviewers, I don’t have queries.

We sincerely thank the reviewer for reviewing the Results section and for having no further queries.

Discussion:

Why 100%? Most of the authors found lower percentage of Staph. Aureus in their researches. The authors mentioned country and production procedure variations. But the commercial cheese production of renowned Brands follows the International standards, even in Bangladesh. In my opinion, authors should establish scientific basis of this finding.

Thank you for this important and insightful comment. The Discussion section has been thoroughly revised to establish a clear scientific basis for the detection rate of S. aureus. We have now incorporated detailed explanations and relevant supporting literature has also been added to strengthen this interpretation.

As all samples exceeded the EU recommended standard, then what could be the authors decision about the commercial cheese in Bangladesh?

Thank you for this valuable observation. We have now clearly stated the authors’ interpretation and decision regarding the microbiological safety of commercial cheese in Bangladesh in the revised Discussion section, emphasizing public health implications and the need for stricter monitoring and enforcement.

Conclusion:

Conclusion became too long. Should be more precise and shorter.

Thank you for your suggestion. The Conclusion section has now been shortened and refined to present only the key findings and implications in a more concise and focused manner.

Line 427: After finishing the whole experiments, showing all findings, why did authors mention “may be’?

Thank you for pointing this out. The use of the term “may be” was unintentional. It has now been removed, and the sentence has been revised to reflect definitive findings based on the experimental results.

………………………………………………………………………………………………………

Dear Reviewer 5:

First, we want to extend our gratitude to you for your valuable comments. May I request you to have your kind attention again on the replies we provided below against your specific comments.

Reviewer: 5

The manuscript entitled “Virulence Determinants and Toxin profile of Methicillin Resistant Staphylococcus aureus from Commercial Cheese in Bangladesh: A public Health Risk” presented the virulence and toxin factors of MRSA from commercial cheese in Bangladesh. The Authors also showed the AMR profile of MRSA by both phenotypic and genotypic approach. The manuscript has great importance in the field of AMR and food safety. However, the manuscript requires some corrections. There are areas of improvement. The knowledge gap should include why the study is different from other studies in Bangladesh. The discussion section has some repetitions. Please check the whole manuscript for English corrections. There is a serious issue in the methodology of bacterial load determination.

We sincerely thank the reviewer for acknowledging the significance of our study on MRSA in commercial cheese in Bangladesh and for the constructive comments. The knowledge gap in the Introduction has been revised to clearly highlight how this study differs from previous research, integrating virulence determinants, toxin profiling, and antimicrobial resistance analysis using both phenotypic and genotypic approaches. The Discussion has been reorganized to remove repetitions and improve clarity, and the bacterial load methodology has been reassessed, clarified, and supported with appropriate references. The manuscript has also been thoroughly edited for English language and readability.

We greatly appreciate your feedback, which has strengthened the manuscript and enhanced its relevance to antimicrobial resistance and food safety.

Below, we provide detailed responses to each point raised:

Title: Why the word “public” starts with the small “p”.

Thank you for pointing this out. The title has been revised to capitalize “Public” appropriately.

Line no. 42: Please add “and” before “Food Safety and Standards Authority India (FSSAI)”.

Thank you for your observation. “and” has been added for correct grammar in Line 42.

Line no. 46: showed a moderate level of resistance. Check the sentence.

Thank you. The antibiotic sensitivity part has been revised for clarity.

The gene name should be Italic. Check the uniformity of gene and species names throughout the manuscript.

Thank you for this important comment. All gene names and species names have been italicized consistently throughout the manuscript.

Please check the whole manuscript for similar minor errors to improve the quality of the manuscript.

We appreciate your suggestion. The entire manuscript has been thoroughly reviewed and corrected for minor grammatical, formatting, and typographical errors.

Line no. 110: Are all the brands from Bangladesh or imported as well?

Thank you for this query. All sampled brands were locally produced in Bangladesh.

Line 134-137: As per standard, the sample amount should be 10 grams. Thereafter, for ten-fold dilution, you must have to maintain 1:10 that is one part sample and 9 parts diluent. However, the Authors have not maintained this ratio. They used one gram sample and 900 microliter (0.9 ml) diluent that is 1: 0.9 ratio. your total volume is approximately 1.9mL. Your dilution factor is 1/1.9, which is approximately 10⁻⁰·²⁸, not 10⁻¹.

It is a serious mistake in the sample preparation and it will misinterpret the findings.

Thank you for highlighting this important issue. We acknowledge the discrepancy. The dilution factor and sample preparation have been revised and corrected following standard protocols (10 g sample with 1:10 dilutions). The methodology section now reflects this correction, and the calculations have been updated accordingly.

Line no. 150-151: Table 01, contains primers of nuc, mecA, and toxin genes.

The supplementary table has been properly referenced and included.

Line no. 156: Table 01. Where is the supplementary table? After Table 1, where is Table 02?

The supplementary table has been properly referenced and included.

Line no. 162-163: The sentence is not clear. Please mention the name of the test with the interpretation criteria.

This part has been revised and written correctly.

Line no. 169-170: Approximate concentration of 1.5*10-8 CFU ml-1.

Corrected.

Line no. 247: total samples.

Corrected.

Line no. 380-382: Please mention the causes of differences in occurrences of MRSA in cheese particularly for Bangladesh.

Thank you. The Discussion has been revised to explain the differences in MRSA occurrence, considering factors such as variations in hygiene practices, production methods, storage, and regulatory implementation in Bangladesh.

………………………………………………………………………………………………………

Dear Reviewer 6:

We sincerely appreciate the time and effort you have devoted to reviewing our manuscript and providing valuable scientific feedback. We kindly request your attention to the responses we have prepared below, addressing each of your specific comments.

Reviewer 6:

Line 95: write the abbreviated form of International Institute for Diarrheal Disease Research within bracket

Thank you for your suggestion. The whole introduction part has been revised to addressed the amendments for better clarity and scientific quality.

Line 132: change the heading 2.3 (Microbiological evaluation of Cheese sample). Aren’t the isolation, identification, molecular detection and antibiotic sensitivity test included in microbiological evaluation? Your writing resembles that only CFU counting represents the microbiological evaluation. You can change the heading as ‘’ total count of Staphylococcus genus’’.

Thank you for pointing this out. The methodology part has been revised regarding the heading and overall workflows for the suitability of the manuscript for the journal.

Line 163: What are the modifications?

Thank you for the query. All modifications made to the methodology have now been explicitly described for clarity.

Line 213: Recheck this heading and make it shorter

All the comments regarding the headings has been addressed.

Line 228: This study performed in Bangladesh. That’s why it is ok to compare the findings with the recommended limit of BFSA. But why the findings are compared to that of Standards Authority of India, and the European Union bypassing other countries like USA, Australia and others?

Thank you for your comment. We compared our findings with FSSAI and EU standards to provide a perspective on the food safety level in Bangladesh relative to widely recognized international benchmarks. These standards are also commonly referenced and followed in our country, making them relevant for contextual comparison.

Line 228-229: Reference?

All relevant references were cited where needed.

Line 286-287: 3.5 Toxin, Virulence, and antibiotic-resistant genes profile of Staphylococcus aureus isolated from cheese: What about SEb and SEd. Clarify their percentage.

Thank you. No isolates were positive for SEb and SEd.

Figure 6: it will be easier to understand if you represent the two pictures of PCR with two different figure names.

Corrected.

Figure 8: same as figure 6

Corrected.

Figure 9: Try to increase the sharpness and clarity of the given diagram.

Thank you. All the diagrams given with the manuscript were prepared with high clarity and sharpness again.

Line 341: Why is a matter of concern?

Addressed properly.

Discussion: line 388-394: Recheck the sequence and organization of the provided information. At first you wrote about the percentage of S. aureus and compared it with the existing data, then wrote about MRSA in the same manner. After that you repeated the S. aureus again. Try to write in more organized way.

Thank you for this observation. The Discussion section has been reorganized to first present the findings for S. aureus, followed by MRSA, avoiding repetition and improving logical flow.

Line 437: is it TEM or blaTEM?

Thank you for pointing this out. The gene name has been corrected to blaTEM.

Overall evaluation: Recheck the whole manuscript again for grammatical errors. The organism’s name and genes name must be in italic form. The headings under different section should be rechecked to make it simpler, smarter and shorter.

We appreciate your suggestion. The entire manuscript has been thoroughly revised for grammar, clarity, and readability. All organism and gene names are now in italic form, and headings have been simplified and shortened for better presentation.

………………………………………………………………………………………………………

Dear Reviewer 7:

We sincerely appreciate your time and valuable feedback and invite you to review our responses to each of your specific comments.

Reviewer 7:

I have reviewed the manuscript "Virulence Determinants and Toxin Profile of Methicillin-Resistant Staphylococcus aureus from Commercial Cheese in Bangladesh: A Public Health Risk" for the first time. The authors have justified or corrected the observations made by reviewers in previous rounds. However, I believe there are still several errors or clarifications that need to be made, particularly in the methods and results.

We sincerely thank the reviewer for taking the time to evaluate our manuscript and for acknowledging the revisions made in response to previous rounds of review. We appreciate your constructive feedback and have carefully addressed all additional comments and clarifications, particularly in the Methods and Results sections, to improve the accuracy, clarity, and overall quality of the manuscript.

Materi

Attachment Submitted filename: Response to Reviewers on latest review.docx

# Peer review: Decision Letter 3

**DOI:** 10.1371/journal.pone.0350222.r007

**Authors:** Abayeneh Girma

31 Mar 2026

PONE-D-24-38694R3

Virulence Determinants and Toxin profile of Methicillin Resistant Staphylococcus aureus from Commercial Cheese in Bangladesh: A public Health Risk

PLOS One

Dear Dr. Hossain,

Thank you for submitting your manuscript to PLOS ONE. After careful consideration, we feel that it has merit but does not fully meet PLOS ONE’s publication criteria as it currently stands. Therefore, we invite you to submit a revised version of the manuscript that addresses the points raised during the review process.

Please submit your revised manuscript by May 15 2026 11:59PM. If you will need more time than this to complete your revisions, please reply to this message or contact the journal office at plosone@plos.org. When you're ready to submit your revision, log on to https://www.editorialmanager.com/pone/ and select the 'Submissions Needing Revision' folder to locate your manuscript file.

Please include the following items when submitting your revised manuscript:

- A letter that responds to each point raised by the academic editor and reviewer(s). You should upload this letter as a separate file labeled 'Response to Reviewers'.
- A marked-up copy of your manuscript that highlights changes made to the original version. You should upload this as a separate file labeled 'Revised Manuscript with Track Changes'.
- An unmarked version of your revised paper without tracked changes. You should upload this as a separate file labeled 'Manuscript'.

If you would like to make changes to your financial disclosure, please include your updated statement in your cover letter. Guidelines for resubmitting your figure files are available below the reviewer comments at the end of this letter.

If applicable, we recommend that you deposit your laboratory protocols in protocols.io to enhance the reproducibility of your results. Protocols.io assigns your protocol its own identifier (DOI) so that it can be cited independently in the future. For instructions see: https://journals.plos.org/plosone/s/submission-guidelines#loc-laboratory-protocols. Additionally, PLOS ONE offers an option for publishing peer-reviewed Lab Protocol articles, which describe protocols hosted on protocols.io. Read more information on sharing protocols at https://plos.org/protocols?utm_medium=editorial-email&utm_source=authorletters&utm_campaign=protocols.

As the corresponding author, your ORCID iD is verified in the submission system and will appear in the published article. PLOS supports the use of ORCID, and we encourage all coauthors to register for an ORCID iD and use it as well. Please encourage your coauthors to verify their ORCID iD within the submission system before final acceptance, as unverified ORCID iDs will not appear in the published article. *Only* the individual author can complete the verification step; PLOS staff *cannot* verify ORCID iDs on behalf of authors.

We look forward to receiving your revised manuscript.

Kind regards,

Abayeneh Girma

Academic Editor

PLOS One

Journal Requirements:

If the reviewer comments include a recommendation to cite specific previously published works, please review and evaluate these publications to determine whether they are relevant and should be cited. There is no requirement to cite these works unless the editor has indicated otherwise.

[Note: HTML markup is below. Please do not edit.]

Reviewers' comments:

Reviewer's Responses to Questions

**Comments to the Author**

1. If the authors have adequately addressed your comments raised in a previous round of review and you feel that this manuscript is now acceptable for publication, you may indicate that here to bypass the “Comments to the Author” section, enter your conflict of interest statement in the “Confidential to Editor” section, and submit your "Accept" recommendation.

Reviewer #7: (No Response)

Reviewer #9: (No Response)

**********

2. Is the manuscript technically sound, and do the data support the conclusions?

The manuscript must describe a technically sound piece of scientific research with data that supports the conclusions. Experiments must have been conducted rigorously, with appropriate controls, replication, and sample sizes. The conclusions must be drawn appropriately based on the data presented.

Reviewer #7: Partly

Reviewer #9: Partly

**********

3. Has the statistical analysis been performed appropriately and rigorously?

Reviewer #7: Yes

Reviewer #9: No

**********

4. Have the authors made all data underlying the findings in their manuscript fully available?

The PLOS Data policy requires authors to make all data underlying the findings described in their manuscript fully available without restriction, with rare exception (please refer to the Data Availability Statement in the manuscript PDF file). The data should be provided as part of the manuscript or its supporting information, or deposited to a public repository. For example, in addition to summary statistics, the data points behind means, medians and variance measures should be available. If there are restrictions on publicly sharing data—e.g. participant privacy or use of data from a third party—those must be specified.

Reviewer #7: Yes

Reviewer #9: Yes

**********

5. Is the manuscript presented in an intelligible fashion and written in standard English?

PLOS ONE does not copyedit accepted manuscripts, so the language in submitted articles must be clear, correct, and unambiguous. Any typographical or grammatical errors should be corrected at revision, so please note any specific errors here.

Reviewer #7: Yes

Reviewer #9: No

**********

6. Review Comments to the Author

Please use the space provided to explain your answers to the questions above. You may also include additional comments for the author, including concerns about dual publication, research ethics, or publication ethics. (Please upload your review as an attachment if it exceeds 20,000 characters)

Reviewer #7: The authors have appropriately corrected or justified almost all of the observations made in the preliminary round of my reviews.

However, they persist in maintaining the phylogenetic analysis and its discussions, which are inappropriate, as already argued in the previous round: "These analyses are inappropriate and should be withdrawn. The fragment considered (233 bp) is very small and highly conserved in S. aureus (I blasted the OR096215 sequence and found nearly 2,000 sequences with 100% identity). The resolution of the phylogenetic tree does not allow for appropriate inferences between identical sequences. Any discussion or conclusion based on this analysis must be withdrawn." Withdrawing this analysis does not detract from the merit of the study. On the contrary, persisting on this point undermines its scientific soundness. I insist on completely withdrawing this analysis (methods, results, and discussions) to avoid inappropriate or unjustified inferences.

Reviewer #9: This manuscript addresses a worthwhile public-health question and has potential value as a regional surveillance study on S. aureus/MRSA in commercial cheese.

However, in its current form, the paper has substantial methodological and reporting weaknesses that limit confidence in the quantitative conclusions. My comments as follows:

1. Unclear unit of analysis / isolate count

The manuscript does not clearly explain how many isolates were actually analyzed. It mentions 72 cheese samples, but many results use denominators that suggest only 12 isolates. It is unclear whether the study used one isolate per sample, per brand, or per positive culture, which makes prevalence and resistance/toxin frequencies hard to interpret.

2. Internal inconsistencies in results

The reported findings conflict across sections. Antibiotic resistance profiles differ between the abstract and Results, and the reported frequencies of biofilm and toxin genes are inconsistent. These are major result discrepancies, not minor wording issues.

3. Problems in microbiological methods

The described pre-enrichment in nutrient broth may be acceptable for detecting presence/absence, but not for quantitative estimation of bacterial load. Since the study also reports CFU/g and compares counts to safety thresholds, the workflow appears methodologically inconsistent. The dilution scheme described is also nonstandard and unclear.

4. Insufficiently robust identification strategy

The identification approach (MSA, Gram stain, catalase/coagulase, nuc PCR) is reasonable for confirming S. aureus, but the manuscript does not clearly explain isolate selection or controls. Given the unusually high claim of 100% S. aureus positivity in all cheese samples, stronger methodological detail is needed.

5. Weak justification for resistance-gene panel

Several screened resistance genes, especially ESBL/carbapenemase genes like CTX-M, CMY, SHV, and NDM-1, are more typical of Gram-negative bacteria and are not well justified in an S. aureus study. Their biological relevance and interpretation are not adequately discussed, and sequencing confirmation is lacking.

6. Overstated toxin conclusions

The study reports toxin genes by PCR, but the manuscript phrases this as if actual toxins were present in the cheese. Gene detection only shows the potential for toxin production, not confirmed toxin expression or toxin presence in the food.

7. Weak statistical analysis

The statistical section lacks a strong analytical framework. Comparing study findings directly to regulatory limits using p-values is not appropriate, and the paper does not provide confidence intervals, clear hypotheses, between-group comparisons, or detailed data presentation.

8. Inadequate phylogenetic analysis

The phylogenetic methods are not described in enough detail to support the claim of identifying a “new strain.” Key details are missing, including number of sequences, gene region, quality control, accession numbers, and reference selection.

**********

7. PLOS authors have the option to publish the peer review history of their article (what does this mean?). If published, this will include your full peer review and any attached files.

If you choose “no”, your identity will remain anonymous but your review may still be made public.

**Do you want your identity to be public for this peer review?** For information about this choice, including consent withdrawal, please see our Privacy Policy.

Reviewer #7: No

Reviewer #9: No

**********

[NOTE: If reviewer comments were submitted as an attachment file, they will be attached to this email and accessible via the submission site. Please log into your account, locate the manuscript record, and check for the action link "View Attachments". If this link does not appear, there are no attachment files.]

To ensure your figures meet our technical requirements, please review our figure guidelines: https://journals.plos.org/plosone/s/figures

You may also use PLOS’s free figure tool, NAAS, to help you prepare publication quality figures: https://journals.plos.org/plosone/s/figures#loc-tools-for-figure-preparation.

NAAS will assess whether your figures meet our technical requirements by comparing each figure against our figure specifications.

# Author comment: Author response to Decision Letter 4

**DOI:** 10.1371/journal.pone.0350222.r008

5 May 2026

Response to Reviewer

PONE-D-24-38694R2

“Virulence Determinants and Toxin Profile of Methicillin-Resistant Staphylococcus aureus from Commercial Cheese in Bangladesh: A Public Health Risk”

Dear Editor

I sincerely appreciate your thoughtful handling of our manuscript. I am also grateful to the reviewers for their valuable insights and constructive feedback, which have greatly strengthened the scientific quality and relevance of our study.

We kindly invite the reviewers to consider our detailed, point-by-point responses to their comments, provided below.

Dear Reviewer 7:

First, we would like to express our gratitude for your time to review our manuscript and for providing scientific criticism on different aspects of our manuscript. May I request you to have your kind attention again on the replies we provided below against your specific comments.

Reviewer: 7

The authors have appropriately corrected or justified almost all of the observations made in the preliminary round of my reviews.

However, they persist in maintaining the phylogenetic analysis and its discussions, which are inappropriate, as already argued in the previous round: "These analyses are inappropriate and should be withdrawn. The fragment considered (233 bp) is very small and highly conserved in S. aureus (I blasted the OR096215 sequence and found nearly 2,000 sequences with 100% identity). The resolution of the phylogenetic tree does not allow for appropriate inferences between identical sequences. Any discussion or conclusion based on this analysis must be withdrawn." Withdrawing this analysis does not detract from the merit of the study. On the contrary, persisting on this point undermines its scientific soundness. I insist on completely withdrawing this analysis (methods, results, and discussions) to avoid inappropriate or unjustified inferences.

We sincerely thank the reviewer for their careful evaluation and for reiterating this important concern. We fully acknowledge the limitations associated with the phylogenetic analysis based on the short (233 bp) and highly conserved fragment, and we appreciate the reviewer’s clarification regarding the lack of discriminatory power and the risk of drawing unsupported inferences.

In response, we have completely removed all components related to the phylogenetic analysis from the revised manuscript, including the corresponding methods, results, figures, and discussion.

………………………………………………………………………………………………………

Dear Reviewer 5:

First, we want to extend our gratitude to you for your valuable comments. May I request you to have your kind attention again on the replies we provided below against your specific comments.

Reviewer: 9

This manuscript addresses a worthwhile public-health question and has potential value as a regional surveillance study on S. aureus/MRSA in commercial cheese.

However, in its current form, the paper has substantial methodological and reporting weaknesses that limit confidence in the quantitative conclusions. My comments as follows:

We sincerely thank the reviewer for recognizing the public health relevance and regional value of our study. We appreciate the constructive feedback regarding the methodological and reporting limitations. In response, we have carefully revised the manuscript to address these concerns, improving the clarity, methodological transparency, and overall robustness of our analyses. We hope the revisions have strengthened confidence in our findings.

Below, we provide detailed responses to each point raised:

1. Unclear unit of analysis / isolate count

The manuscript does not clearly explain how many isolates were actually analyzed. It mentions 72 cheese samples, but many results use denominators that suggest only 12 isolates. It is unclear whether the study used one isolate per sample, per brand, or per positive culture, which makes prevalence and resistance/toxin frequencies hard to interpret.

Thank you for highlighting this important point regarding the clarity of the unit of analysis. In the revised manuscript, we have clearly stated that a total of 120 cheese samples were collected over a six-month period from 12 commercially available brands. All subsequent analyses and interpretations were conducted based on these samples. We have revised the relevant sections to ensure consistency and to avoid any ambiguity regarding isolate numbers and denominators used throughout the manuscript.

2. Internal inconsistencies in results

The reported findings conflict across sections. Antibiotic resistance profiles differ between the abstract and Results, and the reported frequencies of biofilm and toxin genes are inconsistent. These are major result discrepancies, not minor wording issues.

Thank you for carefully identifying these inconsistencies. We acknowledge that the discrepancies in antibiotic resistance profiles, as well as the reported frequencies of biofilm formation and toxin genes, may have caused confusion. In the revised manuscript, all such inconsistencies have been thoroughly checked and corrected. The data have been harmonized across the Abstract, Results, and relevant sections to ensure accuracy, consistency, and clarity.

3. Problems in microbiological methods

The described pre-enrichment in nutrient broth may be acceptable for detecting presence/absence, but not for quantitative estimation of bacterial load. Since the study also reports CFU/g and compares counts to safety thresholds, the workflow appears methodologically inconsistent. The dilution scheme described is also nonstandard and unclear.

Thank you the for this important observation regarding the microbiological methodology. We apologize for the lack of clarity in the previous version. In the revised manuscript, we have clearly explained that each sample was divided into two portions: one portion was used for the detection and isolation of S. aureus and MRSA following enrichment procedures, while the other portion was processed separately for direct microbial enumeration to determine CFU/g without enrichment. We have also revised the description of the dilution and plating procedures to ensure clarity and alignment with standard quantitative methods.

4. Insufficiently robust identification strategy

The identification approach (MSA, Gram stain, catalase/coagulase, nuc PCR) is reasonable for confirming S. aureus, but the manuscript does not clearly explain isolate selection or controls. Given the unusually high claim of 100% S. aureus positivity in all cheese samples, stronger methodological detail is needed.

We appreciate this important comment. We have carefully revised the manuscript to improve the clarity and detail of the identification strategy. We would also like to clarify that the prevalence of S. aureus is not 100% as previously implied; the corrected results show a prevalence of 65%, while MRSA was detected in 30% of the samples. These values have been consistently updated throughout the manuscript. We have thoroughly checked and revised the relevant sections to ensure accuracy, clarity, and consistency.

5. Weak justification for resistance-gene panel

Several screened resistance genes, especially ESBL/carbapenemase genes like CTX-M, CMY, SHV, and NDM-1, are more typical of Gram-negative bacteria and are not well justified in an S. aureus study. Their biological relevance and interpretation are not adequately discussed, and sequencing confirmation is lacking.

Thank you for highlighting this important point. We acknowledge that ESBL and carbapenemase genes such as CTX-M, CMY, SHV, and NDM-1 are primarily associated with Gram-negative organisms, and their biological relevance in S. aureus is not well established. In the revised manuscript, we have carefully reconsidered this aspect and clarified the interpretation of these findings. We have avoided overstatement and emphasized that, if detected, such genes may reflect potential horizontal gene transfer events or methodological limitations, rather than established resistance mechanisms in S. aureus. The discussion has been revised accordingly to ensure scientific accuracy and caution in interpretation.

6. Overstated toxin conclusions

The study reports toxin genes by PCR, but the manuscript phrases this as if actual toxins were present in the cheese. Gene detection only shows the potential for toxin production, not confirmed toxin expression or toxin presence in the food.

We thank the reviewer for this important clarification. We fully agree that the detection of toxin genes by PCR indicates only the genetic potential for toxin production and does not confirm actual toxin expression or presence in the cheese. We would like to clarify that we did not intend to imply confirmed toxin production. In the revised manuscript, we have carefully reviewed and refined the wording throughout to ensure that all statements accurately reflect gene detection rather than toxin presence, thereby avoiding any potential overinterpretation.

7. Weak statistical analysis

The statistical section lacks a strong analytical framework. Comparing study findings directly to regulatory limits using p-values is not appropriate, and the paper does not provide confidence intervals, clear hypotheses, between-group comparisons, or detailed data presentation.

Thank you for highlighting this important issue. We acknowledge that the previous version lacked sufficient analytical depth and clarity. In the revised manuscript, we have strengthened the statistical framework by clearly defining the analytical approach, removing inappropriate comparisons with regulatory limits using p-values, and improving overall data presentation.

8. Inadequate phylogenetic analysis

The phylogenetic methods are not described in enough detail to support the claim of identifying a “new strain.” Key details are missing, including number of sequences, gene region, quality control, accession numbers, and reference selection.

We thank the reviewer for this important observation. We acknowledge that the phylogenetic analysis was not described with sufficient detail to support such claims. In response, and in line with the reviewer’s concerns, we have completely removed all components related to the phylogenetic analysis from the revised manuscript, including the associated methods, results, and discussion. This revision ensures greater scientific rigor and avoids unsupported interpretations.

Dear Reviewers,

We sincerely thank you for your valuable comments and constructive critiques on our manuscript. We greatly appreciate the time and expertise you have invested in evaluating our work. We found your suggestions insightful and highly relevant, and we believe they have significantly improved the quality and suitability of the manuscript for the journal. We have carefully addressed all comments and revised the manuscript accordingly.

Thanks again, and have a good day.

Best regards

Prof. Dr. Ferdaus Mohd Altaf HOSSAIN

Attachment Submitted filename: Response to Reviewers.docx

# Editor report: Decision Letter 4

**DOI:** 10.1371/journal.pone.0350222.r009

**Authors:** Abayeneh Girma

12 May 2026

Virulence Determinants and Toxin profile of Methicillin Resistant Staphylococcus aureus from Commercial Cheese in Bangladesh: A public Health Risk

PONE-D-24-38694R4

Dear Dr. Hossain,

We’re pleased to inform you that your manuscript has been judged scientifically suitable for publication and will be formally accepted for publication once it meets all outstanding technical requirements.

Within one week, you’ll receive an e-mail detailing the required amendments. When these have been addressed, you’ll receive a formal acceptance letter and your manuscript will be scheduled for publication.

An invoice will be generated when your article is formally accepted. Please note, if your institution has a publishing partnership with PLOS and your article meets the relevant criteria, all or part of your publication costs will be covered. Please make sure your user information is up-to-date by logging into Editorial Manager at Editorial Manager® and clicking the ‘Update My Information' link at the top of the page. For questions related to billing, please contact billing support.

If your institution or institutions have a press office, please notify them about your upcoming paper to help maximize its impact. If they’ll be preparing press materials, please inform our press team as soon as possible -- no later than 48 hours after receiving the formal acceptance. Your manuscript will remain under strict press embargo until 2 pm Eastern Time on the date of publication. For more information, please contact onepress@plos.org.

Kind regards,

Abayeneh Girma

Academic Editor

PLOS One

Additional Editor Comments (optional):

Reviewers' comments:

# Editor report: Acceptance letter

**DOI:** 10.1371/journal.pone.0350222.r010

**Authors:** Abayeneh Girma

PONE-D-24-38694R4

PLOS One

Dear Dr. Hossain,

I'm pleased to inform you that your manuscript has been deemed suitable for publication in PLOS One. Congratulations! Your manuscript is now being handed over to our production team.

At this stage, our production department will prepare your paper for publication. This includes ensuring the following:

* All references, tables, and figures are properly cited

* All relevant supporting information is included in the manuscript submission,

* There are no issues that prevent the paper from being properly typeset

You will receive further instructions from the production team, including instructions on how to review your proof when it is ready. Please keep in mind that we are working through a large volume of accepted articles, so please give us a few days to review your paper and let you know the next and final steps.

Lastly, if your institution or institutions have a press office, please let them know about your upcoming paper now to help maximize its impact. If they'll be preparing press materials, please inform our press team within the next 48 hours. Your manuscript will remain under strict press embargo until 2 pm Eastern Time on the date of publication. For more information, please contact onepress@plos.org.

You will receive an invoice from PLOS for your publication fee after your manuscript has reached the completed accept phase. If you receive an email requesting payment before acceptance or for any other service, this may be a phishing scheme. Learn how to identify phishing emails and protect your accounts at https://explore.plos.org/phishing.

If we can help with anything else, please email us at customercare@plos.org.

Thank you for submitting your work to PLOS ONE and supporting open access.

Kind regards,

PLOS ONE Editorial Office Staff

on behalf of

Dr. Abayeneh Girma

Academic Editor

PLOS One
